# Single-cell imaging of T cell immunotherapy responses in vivo

**DOI:** 10.1084/jem.20210314

**Published:** 2021-08-20

**Authors:** Chuan Yan, Qiqi Yang, Songfa Zhang, David G. Millar, Eric J. Alpert, Daniel Do, Alexandra Veloso, Dalton C. Brunson, Benjamin J. Drapkin, Marcello Stanzione, Irene Scarfò, John C. Moore, Sowmya Iyer, Qian Qin, Yun Wei, Karin M. McCarthy, John F. Rawls, Nick J. Dyson, Mark Cobbold, Marcela V. Maus, David M. Langenau

**Affiliations:** 1 Molecular Pathology Unit, Massachusetts General Research Institute, Charlestown, MA; 2 Massachusetts General Hospital Cancer Center, Harvard Medical School, Charlestown, MA; 3 Early Oncology R&D, AstraZeneca, Gaithersburg, MD; 4 Center for Regenerative Medicine, Massachusetts General Hospital, Boston, MA; 5 Harvard Stem Cell Institute, Cambridge, MA; 6 Department of Molecular Genetics and Microbiology, Duke University School of Medicine, Durham, NC

## Abstract

T cell immunotherapies have revolutionized treatment for a subset of cancers. Yet, a major hurdle has been the lack of facile and predicative preclinical animal models that permit dynamic visualization of T cell immune responses at single-cell resolution in vivo. Here, optically clear immunocompromised zebrafish were engrafted with fluorescent-labeled human cancers along with chimeric antigen receptor T (CAR T) cells, bispecific T cell engagers (BiTEs), and antibody peptide epitope conjugates (APECs), allowing real-time single-cell visualization of T cell–based immunotherapies in vivo. This work uncovered important differences in the kinetics of T cell infiltration, tumor cell engagement, and killing between these immunotherapies and established early endpoint analysis to predict therapy responses. We also established EGFR-targeted immunotherapies as a powerful approach to kill rhabdomyosarcoma muscle cancers, providing strong preclinical rationale for assessing a wider array of T cell immunotherapies in this disease.

## Teaser

Xenograft cancer studies using *rag2^Δ/Δ^, il2rga^−/−^* zebrafish allow facile, single-cell imaging of T cell–mediated tumor killing by CAR T cell, BiTE, and APEC immunotherapies and identified EGFR-targeted immunotherapies as an effective treatment for rhabdomyosarcoma muscle cancers.

## Introduction

Chimeric antigen receptor T (CAR T) cells and bispecific T cell engagers (BiTEs) redirect autologous T lymphocytes to kill tumor cells. These immunotherapies have shown exceptional clinical responses in many leukemias and lymphomas (Lee et al., 2015; Lim and June, 2017; Rapoport et al., 2015; Rosenberg and Restifo, 2015; Waldman et al., 2020). However, similar advances have not been made in a large fraction of solid malignancies, largely due to lack of T cell infiltration into the tumor, inefficient in vivo cytotoxicity, and off-target toxicity (Chai et al., 2020). Moreover, cell-based therapies have yet to be fully explored in pediatric solid tumors due in part to the lack of efficacy for these therapies in killing adult solid tumors and lack of preclinical rationale in xenograft models to progress studies into the clinic. This is particularly relevant for rhabdomyosarcoma (RMS), a common pediatric cancer of muscle. RMS is composed of two main subtypes including fusion-positive tumors that harbor *PAX3 *or *PAX7 *fusions with *FOXO1 *and fusion-negative RMSs that have rat sarcoma virus pathway activation (Skapek et al., 2019). Relapse and refractory RMSs have particularly poor prognosis, and new therapies are sorely needed (Yohe et al., 2019). Recently, two pediatric RMS patients with refractory metastatic and recurrent disease were independently treated with HER2^+^ and CD56^+^ CAR T cell therapies, and both had complete disease remission with limited toxicity (Hegde et al., 2020; Jiang et al., 2019). These clinical results suggest a path forward for T cell–mediated therapies in high-risk refractory and metastatic RMS, pending prioritizing CAR T antigen selection and assessing preclinical efficacy in xenograft models.

The advent of ever-increasingly diverse CAR T cells, BiTEs, and additional antibody-based approaches that redirect T cells to engage with tumors has fast outpaced our ability to efficiently test these new therapies in preclinical animal models. For example, new generations of CAR T cells have been engineered to turn on and off CAR T cell responses using chemicals (Giordano-Attianese et al., 2020; Zajc et al., 2020); to express cytokines that increase homing, infiltration, and killing (Adachi et al., 2018; Pegram et al., 2012); and to bind multiple epitopes for increased specificity (Ruella et al., 2016; Wu et al., 2020). Newer innovations such as antibody peptide epitope conjugates (APECs) have also been developed (Millar et al., 2020). These APECs are engineered antibodies that deliver a viral antigen to tumor cells for presentation by HLA-I following proteolytic cleavage by tumor-specific proteases, leading to activation of endogenous CD8^+^ T cell anti-viral immunity and tumor cell killing. This approach has claimed to have superior specificity compared with other antibody-mediated approaches, including BiTEs, by restricting T cell killing to areas within the tumor mass (Millar et al., 2020). Despite the many innovations emerging in the immunoncology field, many T cell immunotherapies have yet to translate into clinical successes, in part attributed to the inefficiencies in preexisting preclinical in vivo modeling approaches to predict poor T cell infiltration into the tumor, low in vivo tumor cell killing, and lack of target specificity—processes that could best be evaluated in vivo and imaged at single-cell resolution.

Here, we report an optically clear adult *rag2^Δ/Δ^, il2rga^−/−^* immunocompromised zebrafish that allows long-term, stable engraftment of human T cells and cancer cells. These mutants were used to engraft fluorescent-labeled human cancers and to quantify responses to CAR T cell, BiTE, and APEC immunotherapies. Single-cell imaging methodologies and high-throughput automated cell counting went on to quantify previously unknown differences between immunotherapies, including quantifying T cell–tumor cell interactions and cytotoxic immune synapse formation. Our work also identified the efficacy of the newly described APEC immunotherapies in redirecting CMV-primed CD8^+^ T cells to kill tumor cells in a wide array of cancer types. This work is important because it provides a strong foundation for moving APECs into clinical evaluation in the future. Lastly, our preclinical xenograft studies identified epidermal growth factor receptor (*EGFR*) T cell immunotherapy as a promising new therapy in a large fraction of pediatric RMSs.

## Results

### *rag2^Δ/Δ^, il2rga^−/−^* zebrafish are a superior xenograft transplantation model

**Figure 1.**
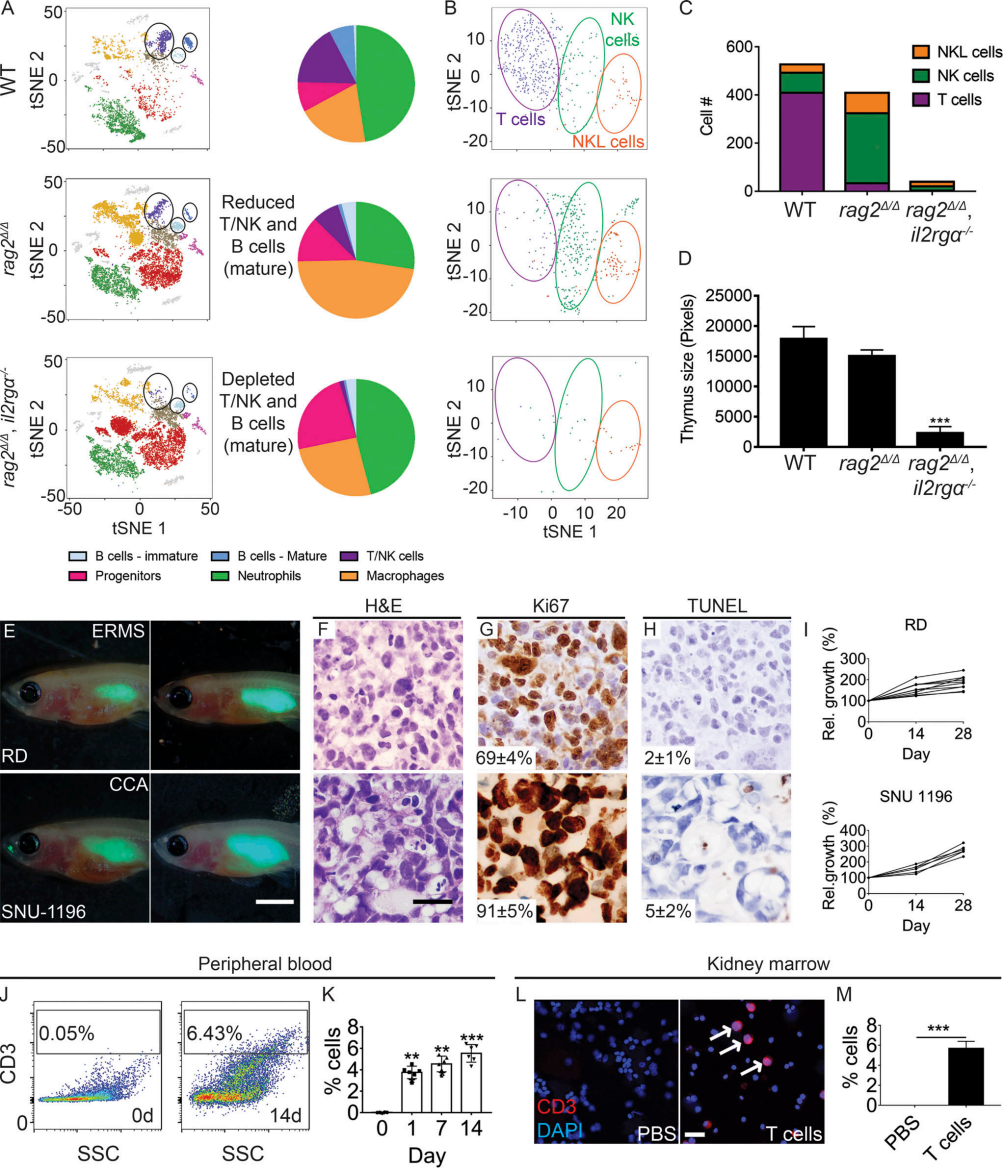
***rag2^Δ/Δ^, il2rga****^−/−^* **zebrafish have reduced T, B, and NK cells and efficiently engraft human cancer and T cells.** **(A)** tSNE visualization and quantitation of single-cell RNA sequencing of the adult kidney marrow. WT, *n* = 4,654 cells; *rag2^Δ/Δ^*, *n* = 9,418 cells; and *rag2^Δ/Δ^, il2rga^−/−^*, *n* = 8,790 cells (*n* = 3 fish/genotype). **(B and C)** tSNE visualization subclustering (B) and quantification (C) of T, NK, and NK-lysin^+^ (NKL) cells within the marrow. **(D)** Histological analysis of thymus size (*n* = 5 fish/genotype). **(E)** Representative images of EGFP^+^ RD embryonal RMS (ERMS) and SNU-1169 cholangiocarcinoma (CCA) cells just after engraftment (0 dpt, left) and at 28 dpt (right). SNU-1169 failed to efficiently engraft into previous immune-deficient zebrafish models. **(F–H)** Histology showing H&E (F), Ki67 (G), and TUNEL (H) staining. *n* ≥ 3 fish/tumor type. **(I)** Kinetics of tumor growth following successful engraftment. **(J–M)** Human CD8^+^ T cells engraft into *rag2^Δ/Δ^, il2rga^−/−^* zebrafish. Flow cytometry analysis of peripheral blood before (left, J) and 14 d after engraftment (right, J). Quantification of human T cells in the peripheral blood (*n* = 5 fish per time point; K). CD3 immunofluorescence staining of kidney marrow cytospins (CD3^+^ cells are red and denoted by arrows; DAPI nuclei staining blue; L) and quantification at 14 d after engraftment (*n* = 6 fish/experimental condition; M). Scale bar equals 0.25 cm (E), 50 µm (F–H), and 10 µm (L). Error bars denote ±SD. **, P < 0.01; ***, P < 0.001, Student's *t* test compared with controls. Rel., relative; SSC, side scatter.

**Figure S1.**
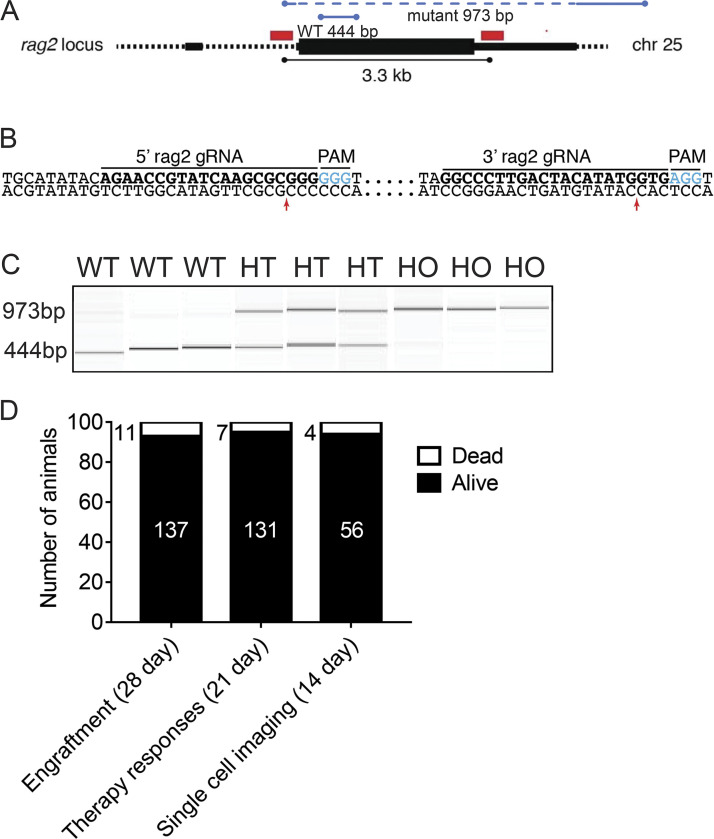
**Creation, genotyping, and viability of *rag2^Δ/Δ^, il2rga****^−/−^* **immunocompromised zebrafish.** **(A)** Schematic of the rag2 locus on chromosome 25, with CRISPR-Cas9 gRNA targets noted by red bars. PCR primers used for genotyping are noted in blue. Deletion mutant and WT-specific PCR fragments are 444 bp and 973 bp, respectively. **(B)** DNA sequences for gRNAs noted by bold lettering and juxtaposed to the target genomic sequence. PAM sequences are noted by blue lettering and predicted cut sites by red arrows. **(C)** QIAxcel gel image of amplified PCR fragments from WT, heterozygous *rag2^Δ/+^* (HT), and homozygous *rag2^Δ/Δ^* (HO) fish. **(D)** Survival statistics for *rag2^Δ/Δ^, il2rga^−/−^* zebrafish used in general engraftment studies shown in Fig. 1 and Fig. S2 (89.0% survival); in assessing immunotherapy responses following IP engraftment and injection of T cell products (94.9% survival); and in quantitating immune cell function at single-cell resolution following engraftment into the periocular musculature, IP injection with T cell products, and serial confocal imaging (93.3% survival). Animals largely died due to handling during imaging procedures associated with anesthesia, with none succumbing to overt infection during experiments outlined in this work (*n* = 984 animals).

**Figure S2.**
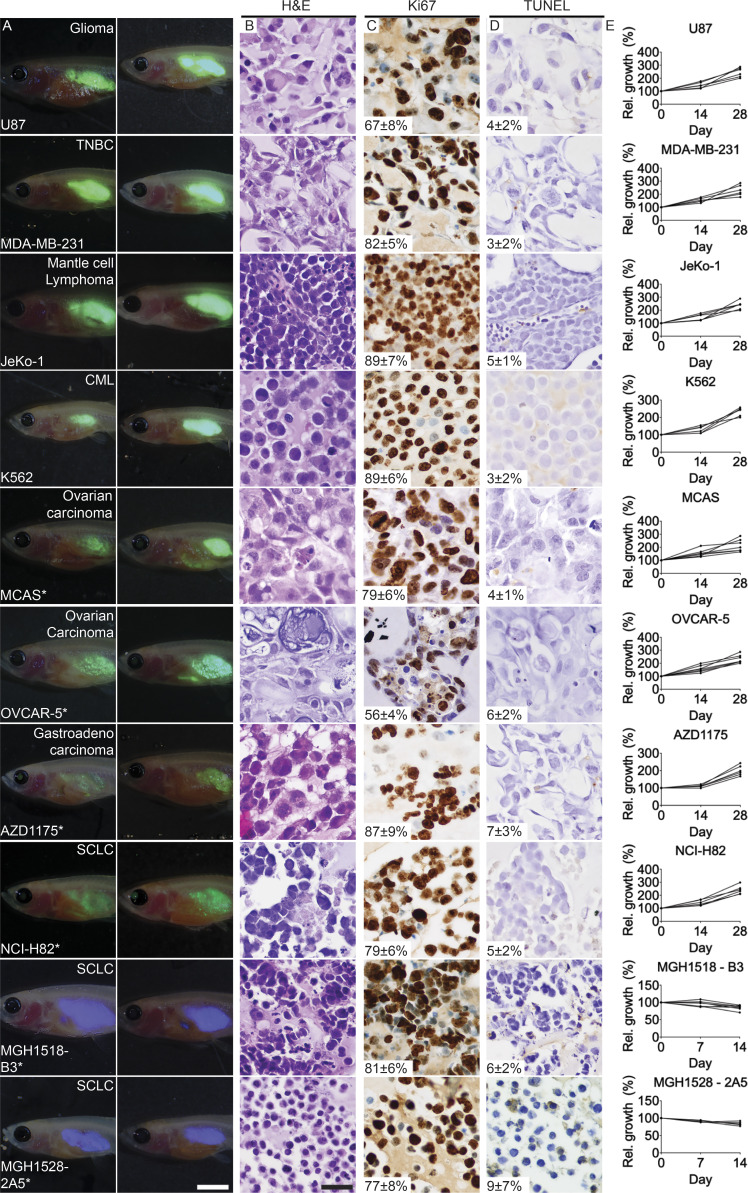
**Engraftment of human cancer cells into *rag2^Δ/Δ^, il2rga****^−/−^* **zebrafish.** **(A)** Representative merged fluorescence and brightfield images of EGFP^+^ human cancers grown in *rag2^Δ/Δ^, il2rga^−/−^* zebrafish. Images shown just after engraftment (0 dpt; left) and at 28 dpt (right). Tumor type is noted in upper right and cell line name in lower left in leftmost panels. TNBC, triple negative breast cancer; CML, chronic myeloid leukemia; SCLC, small cell lung cancer. Tumor growth was assessed by relative GFP intensity multiplied by 2D pixel volume, with exception of PDX tumors that were stained with CFSE. In these PDX engrafted tumors, retention of blue CFSE denotes successful long-term engraftment. **(B)** H&E-stained sections of engrafted tumors. **(C)** Ki67 IHC to assess tumor cell proliferation. **(D)** TUNEL staining to assess apoptotic cells. Average percentage cells ±SD noted (*n* ≥ 3 fish/tumor type; B–D). **(E)** Quantification of relative (Rel.) growth of EGFP^+^ human cancer cells following successful engraftment into individual *rag2^Δ/Δ^, il2rga^−/−^* zebrafish. Scale bars equal 0.25 cm (A) and 50 µm (B–D). * denotes cell lines that did not efficiently engraft in previous immune-deficient zebrafish models.

**Figure S3.**
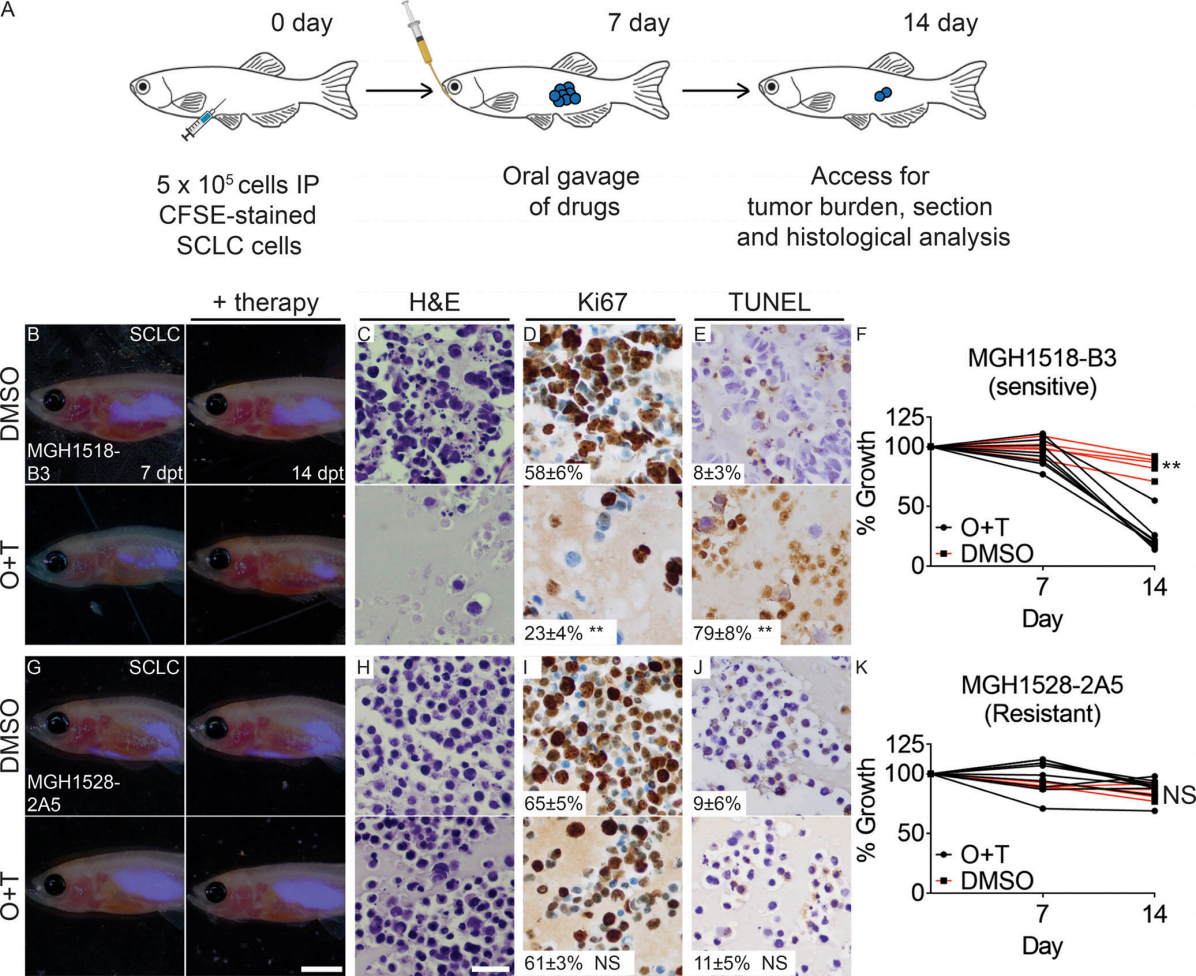
**Accurately predicting olaparib and temozolomide therapy responses in PDXs of small cell lung cancer.** **(A)** Schematic of experimental design. **(B–F)** Xenograft studies of therapy-responsive MGH1518 B3 patient-derived SCLC (*n* ≥ 5 fish/arm). **(G–K)** Xenograft studies of therapy-resistant MGH1528 2A5 patient-derived small cell lung cancer (SCLC; *n* ≥ 5 fish/arm). Merged fluorescence and brightfield images of *rag2^Δ/Δ^, il2rga^−/−^* animals engrafted with CFSE-stained tumor cells before therapy (7 dpt) and after DMSO control (top panels) or combination treatment (bottom panels; B and G). Histological analysis of engrafted tumors showing H&E (C and H), Ki67 (D and I), and TUNEL staining (E and J). Quantification of relative tumor cell growth following control or combination therapy (F and K; *n* ≥ 5 animal/treatment arm). Percentage of cells ±SD noted (*n* = 3 fish/tumor type; D, E, I, and J). **, P < 0.01, Students *t* test. Scale bar equals 0.25 cm (B and G) and 50 µm (C–E and H–J). O&T, olaparib and temozolomide.

Here, we generated a new mutant line of optically clear zebrafish that deletes the entirety of the 3.1-kb recombination activating gene 2 (*rag2*; Fig. 1 and Fig. S1) and inactivates the interleukin 2 receptor gamma α (*il2rga*). These new *rag2^Δ/Δ^, il2rga^−/−^* animals are severely immune deficient and lack most mature B, T, and natural killer (NK) cells (Fig. 1, A–D), consistent with the reported immune profile of C;129S4-Rag2tm1.1Flv Il2rgtm1.1Flv/J mice (Goldman et al., 1998). Compound mutant *rag2^Δ/Δ^, il2rga^−/−^* animals were generated at the expected Mendelian ratios, were viable into adulthood, and robustly engrafted a wider array of human tumors than our previously described *prkdc^−/−^, il2rga^−/−^* model (Fig. 1, E–I; and Fig. S2). As expected, the histopathology and cell morphological features of engrafted tumors were similar to those of patient tumors and those grown in *NOD.Cg-Prkdc^scid^ Il2rg^tm1Wjl^/SzJ*  (NSG) mice (Fig. 1, E–I; and Fig. S2). We were also able to predict patient-derived xenograft (PDX) responses to combination olaparib Poly (ADP-ribose) polymerase inhibitor and temozolomide DNA–damaging agent in small cell lung cancer using clinically relevant, oral dosing (Fig. S3). Finally, we engrafted normal human CD8^+^ T cells into *rag2^Δ/Δ^, il2rga^−/−^* animals. These CD8^+^ T cells remained in the circulation and colonized the kidney marrow of engrafted animals. In total, up to 6% of the peripheral blood and kidney marrow was composed of human CD8^+^ cells by 14 d post transplantation (dpt; Fig. 1, J–M). Our results establish the *rag2^Δ/Δ^, il2rga^−/−^* as an improved xenograft transplantation model with specific utility in engrafting human T cells.

### Assessing CAR T cell and BiTE immunotherapy responses in *rag2^Δ/Δ^, il2rga^−/−^* zebrafish

**Figure 2.**
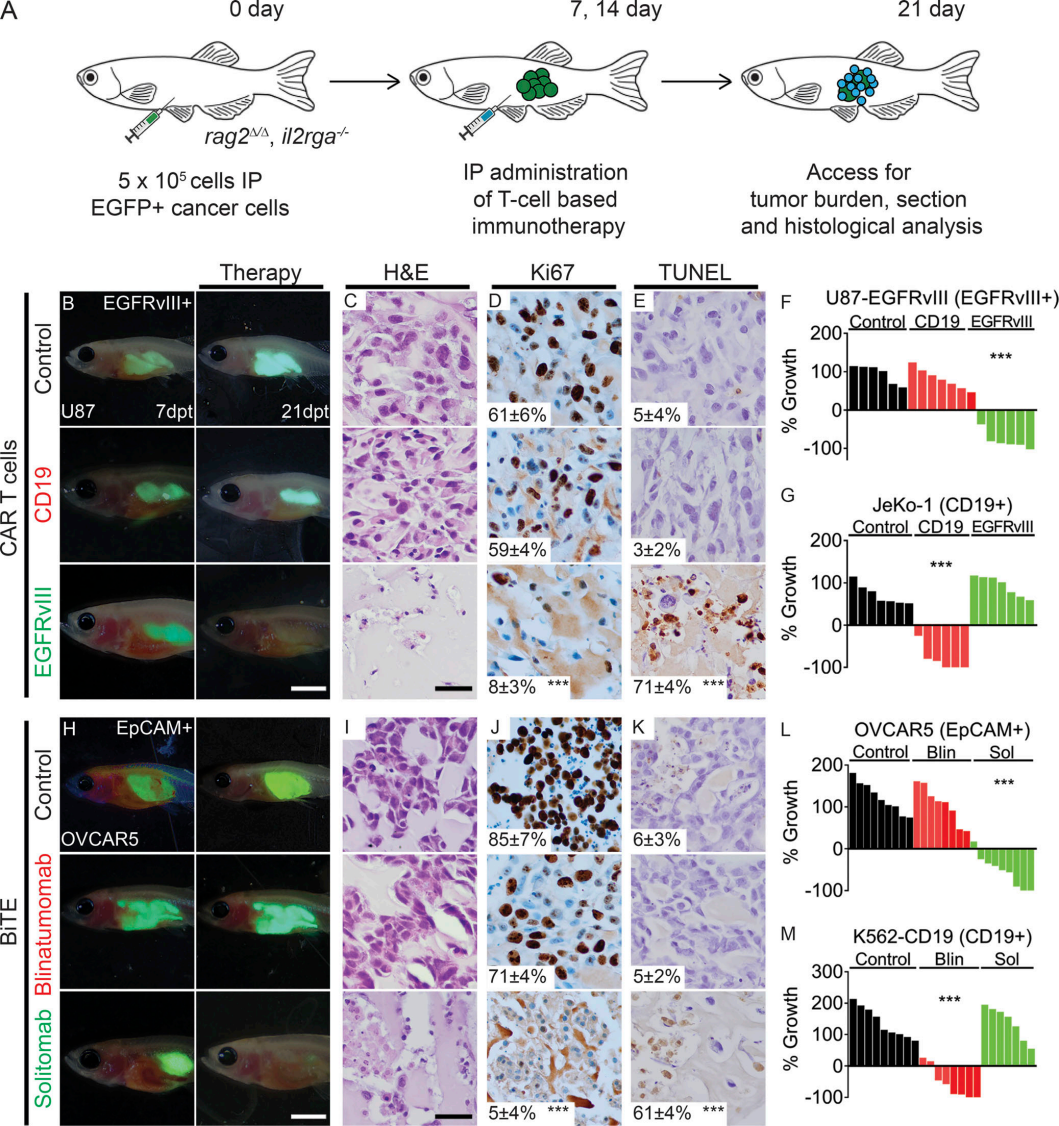
**Preclinical evaluation of CAR T cell and BiTE immunotherapies using xenografts grown in *rag2^Δ/Δ^, il2rga****^−/−^* **zebrafish.** **(A)** Schematic of experimental design. **(B–G)** CAR T immunotherapy. **(H–M)** BiTE immunotherapy. **(B–F)** *rag2^Δ/Δ^, il2rga^−/−^* animals engrafted with GFP^+^ U87 glioma cells that coexpress EGFRvIII and administered CFSE-labeled untransduced control T cells, CD19 CAR T cells, or EGFRvIII CAR T cells (*n* ≥ 6 fish/arm). **(G)** Quantification of relative tumor cell growth following CAR T cell administration in EGFP^+^ JeKo-1 B cell lymphomas that express endogenous CD19. **(H–L)** *rag2^Δ/Δ^, il2rga^−/−^* animals engrafted with EGFP^+^ OVCAR-5 ovarian cancer cells that endogenously express EpCAM and monitored for tumor regressions following injection of CD8^+^ T cells and either EpCAM control antibody, CD19-CD3 blinatumomab, or EpCAM-CD3 solitomab BiTEs (*n* ≥ 7 fish/arm). **(M)** Quantification of relative tumor cell growth following BiTE administration in K562 CML cells engineered to express EGFP and CD19. Merged fluorescence and brightfield images of engrafted animals at 7 dpt (before treatment; left panels in B and H) and 21 dpt (after immunotherapy; right panels in B and H). H&E- (C and I), Ki67- (D and J), and TUNEL-stained (E and K) sections with quantification noted ±SD (*n* = 3 fish/arm). Waterfall plots quantifying relative tumor growth at 21 dpt with the epitope expressed noted in parenthesis (F, G, L, and M). ***, P < 0.001, Student’s *t* test compared with control treated fish. Scale bar equals 0.25 cm (B and H) and 50 µm (C–E and I–K). Blin, blinatumomab; CML, chronic myeloid leukemia; Sol, solitomab.

**Figure S4.**
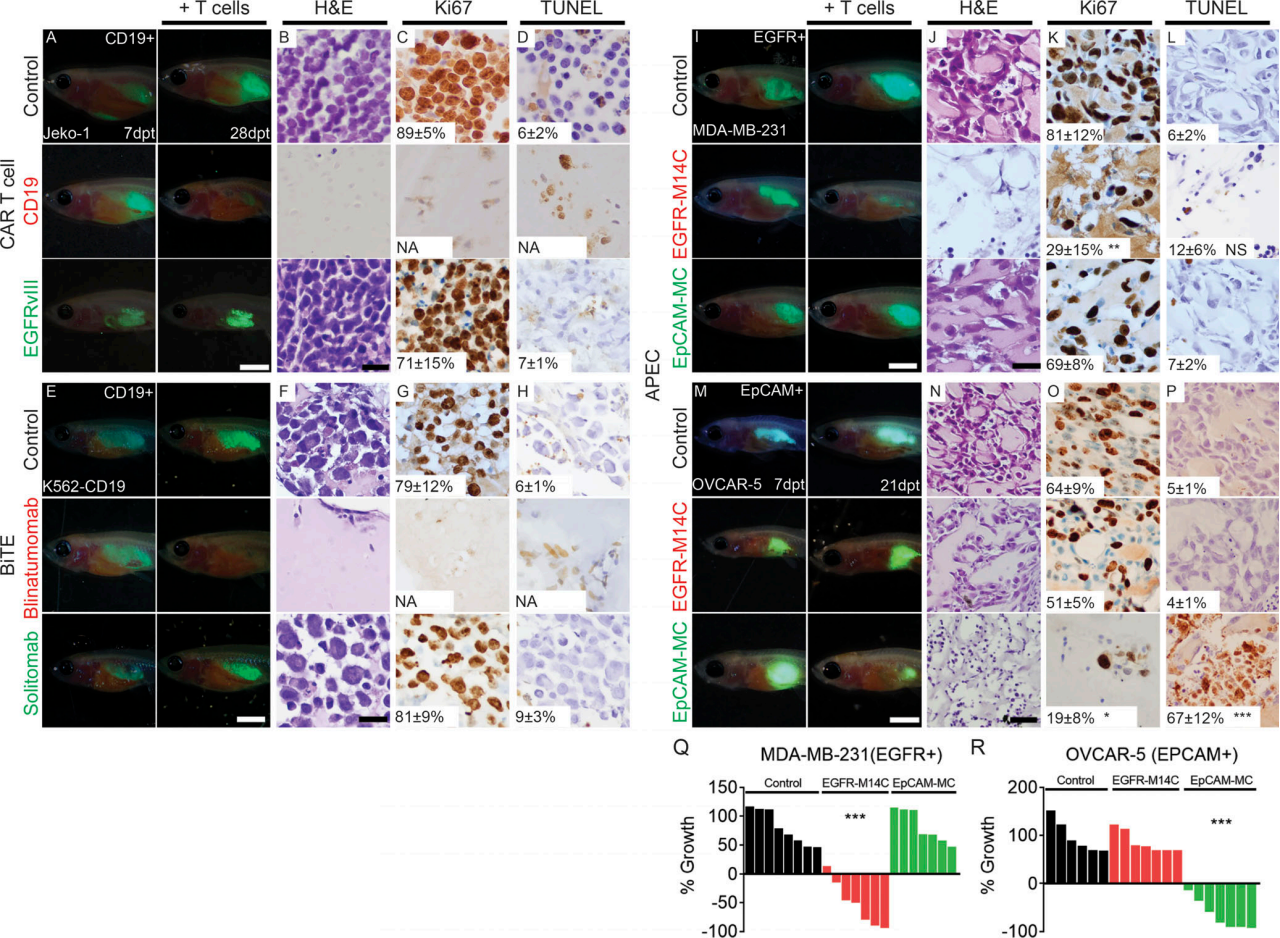
**Preclinical evaluation of CAR T cell, BiTE, and APEC immunotherapies following IP engraftment of human cancers into *rag2^Δ/Δ^, il2rga^−/−^* zebrafish.** **(A–D)** *rag2^Δ/Δ^, il2rga^−/−^* animals engrafted with EGFP^+^ JeKo-1 B cell lymphoma that express endogenous CD19 and then were followed for tumor regressions after injection of untransduced control T cells, CD19 CAR T cells, or EGFRvIII CAR T cells. **(E–H)** *rag2^Δ/Δ^, il2rga^−/−^* animals engrafted with EGFP^+^ K562 CML cells that express exogenous CD19 and were monitored for tumor regressions following injection of CD8^+^ T cells and either EpCAM control antibody, CD19/CD3 blinatumomab, or EpCAM/CD3 solitomab BiTEs. **(I–L and Q)** *rag2^Δ/Δ^, il2rga^−/−^*, animals engrafted with EGFP^+^ MDA–MBA-231 cancer cells that endogenously express the EGFR epitope and then followed for tumor regression and after injection of CMV-specific CD8^+^ T cells and either EpCAM antibody (control), EGFR-M14C, or EpCAM-MC (IP administration of therapies was completed at 7 dpt and 14 dpt). **(M–P and R)** *rag2^Δ/Δ^, il2rga^−/−^* animals engrafted with EGFP^+^ OVCAR-5 ovarian cancer cells that endogenously express the EpCAM epitope and then followed for tumor regression and after injection of CMV-specific CD8^+^ T cells and either EpCAM antibody (control), EGFR-M14C, or EpCAM-MC (IP administration of therapies was completed at 7 dpt and 14 dpt; *n* ≥ 7 fish/arm). Whole-animal imaging of engrafted animals at 7 dpt (before T cell immunotherapy, left panels in A, E, I, and M) and 28 dpt (after therapy, right panels in A, E, I, and M). H&E- (B, F, J, and N), Ki67- (C, G, K, and O), and TUNEL-stained (D, H, L, and P) sections at 28 dpf (days post-fertilization), with quantification noted ±SD (*n* = 3 fish/arm). Scale bar equals 0.25 cm (A, E, I, and M) and 50 µm (B–D, F–H, J–L, and N–P). NA, not accessible due to lack of tumor cells detected on section. Sections are stained with H&E (B and G), Ki67 (C and H), and TUNEL (D and I). **(Q)** Quantification of relative tumor cell growth following APEC administration in EGFP^+^ MDA–MBA-231 TNBC cells. **(R)** Quantification of relative tumor cell growth following APEC administration in EGFP^+^ OVCAR-5 ovarian cancer cells. The average percentage of positive cells ±SD is noted (*n* ≥ 3 fish). *, P < 0.05; **, P < 0.01; ***, P < 0.001 by Student’s *t* test. Scale bar equals 0.25 cm (A, E, I, and N) and 50 µm (B–D, F–H, J–L, and O–Q). CML, chronic myeloid leukemia; TNBC, triple negative breast cancer.

Next, we assessed the utility of *rag2^Δ/Δ^, il2rga^−/−^* zebrafish for preclinical modeling of T cell immunotherapy responses. Here, we tested a wide range of clinically relevant T cell immunotherapies that are either U.S. Food and Drug Administration approved (i.e., CD19 CAR T cells and blinatumomab) or under clinical evaluation in open trials (i.e., EGFRvIII CAR T cells and solitomab). First, we engrafted GFP-expressing EGFRvIII^+^ U87 glioma into the peritoneal cavity of *rag2^Δ/Δ^, il2rga^−/−^* mutant animals. Engrafted animals were administered (i) untransduced T cells, (ii) nontargeted CD19 CAR T cells, or (iii) EGFRvIII-targeted CAR T cells on days 7 and 14 after tumor cell engraftment (Fig. 2 A). Notably, only EGFRvIII-targeted CAR T cells efficiently killed glioma tumors (*n* = 6 animals/arm, P < 0.001, Student’s *t* test), while tumor regressions were not observed in animals injected with untransduced or nontargeted CD19 CAR T cells (Fig. 2, B and F). Histopathology analysis and Tdt-mediated dUTP-biotin nick end labeling (TUNEL) staining confirmed on-target tumor cell killing by EGFRvIII-targeted CAR T cells but not control T cells (Fig. 2, C–E). Similarly, CD19^+^ JeKo-1 B lymphomas were killed only following administration of CD19-specific CAR T cells, but not untransduced or nontargeted EGFRvIII CAR T cells (Fig. 2 G; and Fig. S4, A–D). These results are similar to those of xenograft studies performed in NSG mice (Fraietta et al., 2016; O’Rourke et al., 2017) and credential the zebrafish model for accurately assessing CAR T cell responses in vivo.

Next, we investigated the in vivo therapy responses and specificity to BiTE immunotherapy. BiTEs are artificial, bispecific monoclonal antibodies that contain two single-chain variable fragments, one that binds T cells through contact with the CD3 receptor while the other binds to a tumor-specific molecule, redirecting endogenous cytotoxic T cells to engage and kill cancer cells. Here, animals were engrafted with GFP-expressing EpCAM^+^ OVCAR-5 ovarian carcinoma cells or chronic myelogenous leukemia K562 cells engineered to stably express human CD19 (K562-CD19). After 7 d of engraftment, animals were coinjected with (i) solitomab (EpCAM/CD3) BiTEs, (ii) blinatumomab (CD19/CD3) BiTEs, or (iii) EpCAM control antibody along with human CD8^+^ T cells weekly. Tumor regressions were only observed within animals administered target-specific BiTEs by 21 d after engraftment (P < 0.001, Student’s *t* test; *n* = 8 animals/arm; Fig. 2, H–M and Fig. S4, E–H). Together, these experiments highlight the exquisitely high degree of target specificity for each BiTE and the relative ease of reading out these tumor responses in live zebrafish when compared with mouse xenograft studies (Brischwein et al., 2006; Mølhøj et al., 2007).

### Single-cell imaging of T cell immunotherapy migration and infiltration in vivo

**Figure 3.**
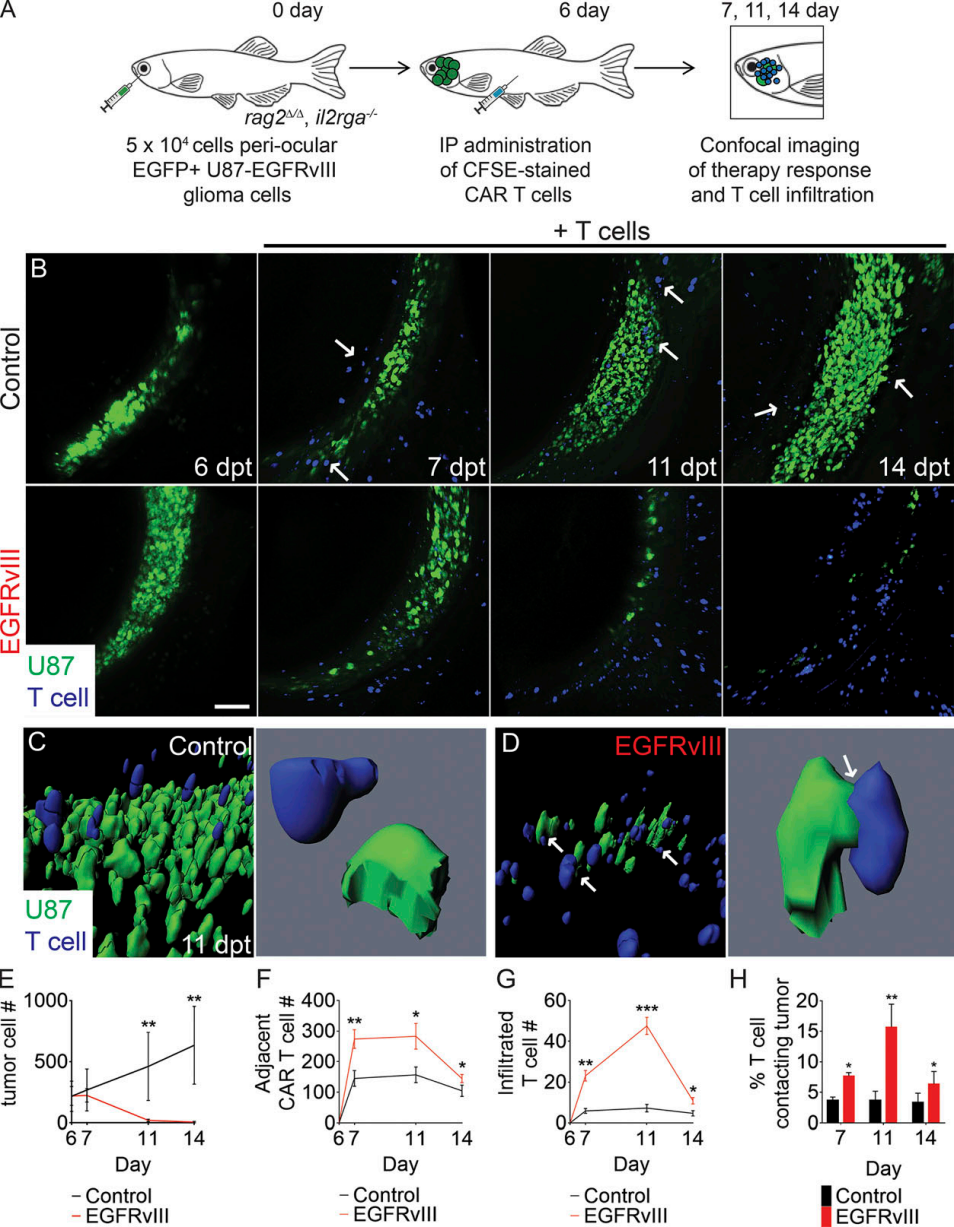
**Dynamic single-cell imaging of EGFRvIII CAR T cell infiltration and tumor cell engagement in human glioma xenografts**. **(A)** Schematic of experimental design. **(B)** Serial imaging of animal engrafted with U87 glioma cells engineered to express EGFP and EGFRvIII into the periocular muscle and imaged before (6 dpt) or after IP injection of CFSE-labeled untransduced T cells or EGFRvIII CAR T cells. Arrows show control T cells aligned on the periphery of the tumor mass. **(C and D)** 3D modeling of control (C) and EGFRvIII CAR T–treated animals (D) at 11 dpt. Arrows show T cells that directly contact tumor cells. **(E)** Tumor growth (*n* = 5 fish/experimental arm, 0.1 mm^3^ volume). **(F and G)** Quantification of CAR T cell migration adjacent to the tumor (F) and infiltrated into the tumor (G; *n* = 5 animals/experimental arm, 0.1 mm^3^ volume). **(H)** Quantification of CAR T cells directly contacting tumor cells over time. *, P < 0.05; **, P < 0.01; ***, P < 0.001, Student’s *t* test. Scale bar equals 100 µm (B) and 10 µm (C and D; lower magnification images). Error bars denote ±SD (E–H).

We next investigated the kinetics of CAR T cell infiltration and engagement when encountering antigen-specific tumor cells using live animal, single-cell imaging. Specifically, 5 × 10^4^ GFP-expressing U87 EGFRvIII^+^ glioma cells were engrafted into the superficial orbital musculature of *rag2^Δ/Δ^, il2rga^−/−^* animals. This site is easily assessable for confocal microscopy imaging (Yan et al., 2019; Yan et al., 2020). Following engraftment for 6 d, *rag2^Δ/Δ^, il2rga^−/−^* animals were intraperitoneally (IP) injected with a single dose of nontargeted control T cells or EGFRvIII CAR T cells (5 × 10^5^ cells; Fig. 3 A). T cells were ex vivo labeled with the CFSE cell-permeable dye prior to implantation. Although CFSE intensity decreases by half at each cell division, published studies using engraftment into NSG mice have demonstrated that human T cells undergo only one or two divisions in the first 7 dpt, permitting facile imaging of stained T cells in vivo using confocal microscopy (Quah et al., 2007; Wong and Pamer, 2001). Using this approach, we were able to quantify migration to regions adjacent the tumor by 24 h after injection with both nontargeted and EGFRvIII CAR T cells (Fig. 3, B and F; and Video 1; *n* = 5 animals per condition). Yet, only EGFRvIII CAR T cells could efficiently enter the tumor mass and kill tumor cells by 5 d after therapy administration (Fig. 3, B–E; P < 0.01, Student’s *t* test).

**Video 1.** **3D modeling of CAR T cell responses to U87-EGFRvIII glioma tumors grown in a *rag2^Δ/Δ^, il2rga****^−/−^* **zebrafish.** Control experiments used untransduced T cells (blue) and were compared with animals injected with EGFRvIII CAR T cells (blue). Tumor cells were labeled with GFP. Animals were imaged at 11 d after engraftment (5 d after infusion with T cell products). The control experiments show untransduced T cells lining the tumor periphery and failing to infiltrate or engage tumor cells. By contrast, EGFRvIII CAR T cells robustly infiltrated tumor and engaged with glioma tumor cells. Note the reduction in overall tumor burden in animals injected with EGFRvIII CAR T cells. 1× playback speed.

**Figure S5.**
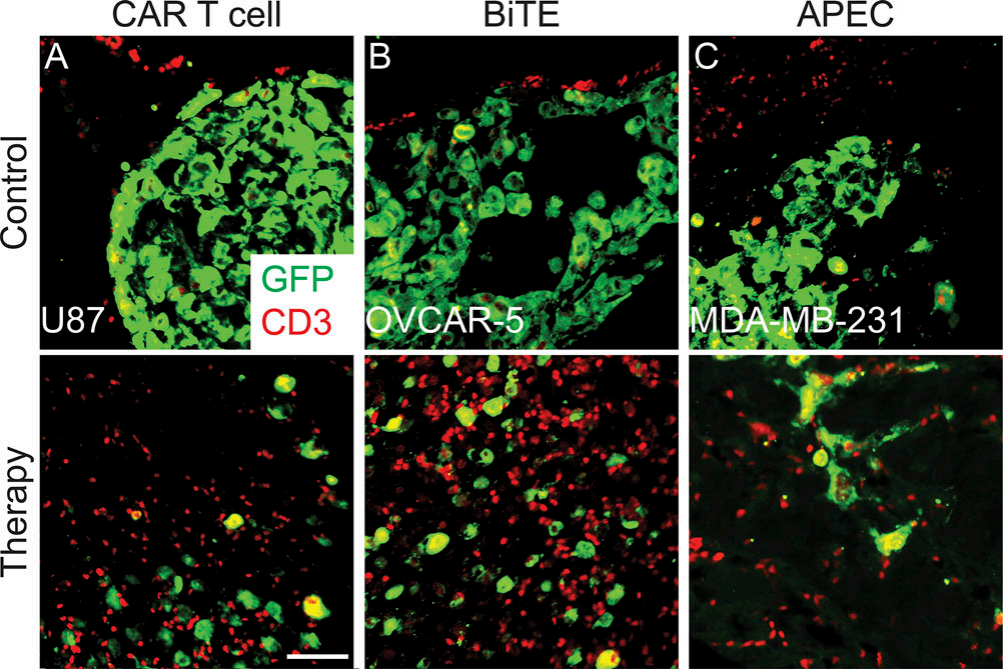
**IHC validation of T cell infiltration into engrafted tumors following immunotherapy.** **(A)** IHC analysis of *rag2^Δ/Δ^, il2rga^−/−^* animals engrafted with U87 glioma cells engineered to express EGFP and EGFRvIII and then assessed for CD3^+^ T cell infiltration at 21 dpt. Animal receiving untransduced control T cells (top) or CD19 CAR T cell therapy (bottom) by IP injection on 7 dpt and 14 dpt. **(B)** *rag2^Δ/Δ^, il2rga^−/−^* animals engrafted with EGFP^+^ OVCAR-5 ovarian cancer cells that express endogenous EpCAM and monitored for CD3^+^ T cell infiltration at 21 dpt (IP therapy delivered on 7 dpt and 14 dpt). Animal received injection of CD8^+^ T cells along with EpCAM control antibody (top) or EpCAM-CD3 solitomab BiTE therapy (bottom). **(C)** Fish engrafted with EGFP^+^ breast cancer MDA–MB-231 cells that endogenously express EGFR and monitored for CD3^+^ T cell infiltration at 21 dpt. Animals were coadministered either EGFR control antibody (top) or EGFR-M14C along with CMV-specific T cells (bottom, IP injected on day 7 and 14 d). Scale bar is 50 µm.

High-magnification 3D volumetric modeling revealed remarkable differences in cell behavior. EGFRvIII CAR T cells rapidly infiltrated into the tumor and subsequently engaged with tumor cells by 5 d after T cell injection (Fig. 3, C–H; and Video 1). By contrast, nontransduced T cells migrated to the transplant site (Fig. 3 C) but did not infiltrate the tumor and rather aligned along the peripheral edge (*n* = 718 of 750 cells analyzed across all time points, *n* = 5 animals; Video 1). Similar differences in CAR T cell infiltration and additional T cell–mediated immunotherapies were observed on sectioning of animals at the end of the experiment and costaining of samples with human CD3 and the GFP antibody (Fig. S5). Finally, we were able to directly quantify the number of T cell–tumor cell interactions over time, showing initial engagement of CAR T cells with tumor cells by just 1 d after injection and maximal CAR T cell–tumor cell contact and subsequent tumor cell killing by 5 d after CAR T infusion (Fig. 3 H). By 8 d after immunotherapy, CAR T cell engagement was reduced, likely reflecting near-complete ablation of tumor cells by this time point (Fig. 3, E and H). In total, these experiments provide a detailed understanding of the kinetics of CAR T cell migration to the tumor site, infiltration, engagement, and subsequent tumor cell killing in vivo*.*

### Real-time quantification of BiTEs induced cell killing in vivo

**Figure 4.**
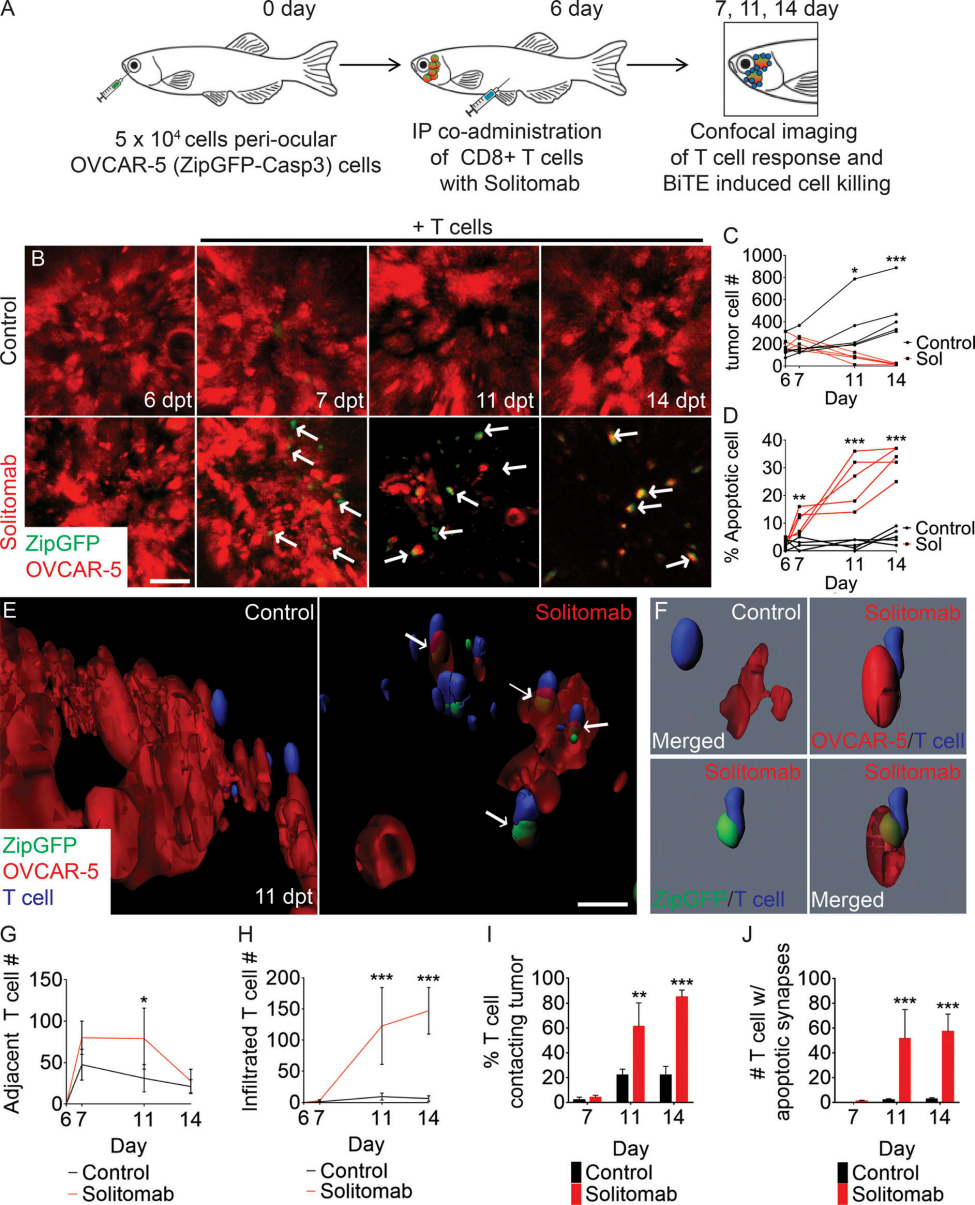
**Single-cell quantitation of apoptotic immune synapse formation following BiTE immunotherapy in ovarian carcinoma.** **(A)** Schematic of experimental design. **(B)** Serial imaging of animals engrafted with OVCAR-5 cells engineered to express mCherry and ZipGFP-Casp3 before (6 dpt) and after IP injection of CFSE-labeled CD8^+^ T cells administered with either EpCAM antibody (control) or EpCAM/CD3 solitomab (7, 11, and 14 dpt; arrows denote apoptotic cells). **(C)** Quantification of tumor cell numbers in engrafted animals over time. **(D)** Quantification of apoptotic cells over time. **(E)** 3D modeling showing control EpCAM antibody (left) or CD3/EpCAM solitomab (right) imaged at 11 dpt (white arrows denote apoptotic cells engaged with T cells). **(F)** Single-cell renderings showing immune synapse formation in real time. Control (top left panel) and experiment (top right and bottom panels). **(G and H)** T cell migration to sites adjacent to the tumor (G) and infiltrated into the tumor mass (H; 0.1 mm^3^ volume). **(I)** Quantification of the percentage of T cells contacting tumor cells. **(J)** Number of tumor-infiltrating T cells that contain apoptotic synapses with tumor cells. 0.0125 mm^3^ volume. *, P < 0.05; **, P < 0.01; ***, P < 0.001, Student’s *t* test. Scale bar equals 10 µm (B and E). *n* = 5 fish/experimental arm for all analyses shown. Error bars denote ±SD. Sol, solitomab.

Next, we developed a facile single-cell imaging platform to rapidly assess on-target cell killing in vivo and image apoptotic immune synapse formation as an early end point analysis of cell killing. Specifically, we engineered EpCAM^+^ OVCAR-5 ovarian carcinoma cells to stably express both mCherry and the ZipGFP-Casp3 apoptotic cell reporter, a GFP variant that only emits fluorescence upon Casp3 cleavage of an inhibitory protease sequence inserted between β1-10 and β11 barrel of protein (To et al., 2016). mCherry^+^/ZipGFP-Casp3^+^ OVCAR-5 cells were engrafted into the periocular musculature and 6 d later IP injected with 5 × 10^5^ CD8^+^ CFSE dye–labeled T cells and either (i) 50 µg/kg control EpCAM antibody or (ii) EpCAM/CD3 solitomab (Fig. 4 A). Solitomab-treated animals had decreased tumor burden over time (Fig. 4 C) and exhibited a significant increase in ZipGFP-Casp3–labeled apoptotic cells starting at 24 h after therapy compared with control EpCAM antibody–treated fish (P < 0.01, Student’s test; Fig. 4, B and D). Elevated numbers of ZipGFP-Casp3^+^ cells were observed by 24 h after treatment, preceding the overall reduction in tumor cells that was observed at 4 and 7 d after treatment with EpCAM/CD3 solitomab, suggesting that BiTE-induced cell killing required >24 h to reach maximal efficiency (Fig. 4, C and D).

BiTE-induced cytotoxicity requires physical contact of T cells and tumor cells, forming apoptotic immunological synapse and release of cytolytic granules by T cells in vitro (Roda-Navarro and Álvarez-Vallina, 2020). As expected, solitomab treatment led to high T cell migration and infiltration into the tumor (Fig. 4, E, G, and H). 3D modeling of CD8^+^ T cells with ZipGFP-Casp3^+^ tumor cells allowed in vivo imaging of T cell–tumor cell contact and quantitation of apoptotic immunological synapses in vivo (Fig. 4 F). 83% of all CD8^+^ T cells were in direct contact with tumor cells after 7 d of solitomab treatment compared with 24% in EpCAM antibody–treated controls (P < 0.01, Student’s *t* test; Fig. 4 I and Video 2). Solitomab treatment also led to higher rates of apoptotic immunological synapse formation by 4 and 7 d of treatment (Fig. 4 J). In addition to high-resolution imaging of cell killing in vivo, these experiments rapidly assessed therapy-induced cell killing that could first be detected 4 d after BiTEs administration, providing a robust and fast assay for determining on-target in vivo killing.

**Video 2.** **3D modeling of BiTE responses to OVCAR-5 ovarian carcinoma cells grown in *rag2^Δ/Δ^, il2rga****^−/−^* **zebrafish.** OVCAR-5 cells were engineered to coexpress mCherry and the apoptosis ZipCasp-GFP reporter. Control experiments used infusion of EpCAM antibody along with CD8^+^ T cells (blue) and were compared with animals injected with solitomab EpCAM/CD3 BiTEs along with CD8^+^ T cells (blue). Animals were imaged at 11 d after engraftment (5 d after infusion with T cell products). The control experiments reveal only minimal tumor cell apoptosis and T cell engagement with tumor. By contrast, animals injected with EpCAM/CD3 BiTEs and CD8^+^ T cells led to the formation of cytotoxic immune synapses and robust tumor cell apoptosis. Live tumor cells express only mCherry^+^, while dying cells are ZipCasp-GFP^+^. 1× playback speed.

### Assessing target specificity of APEC immunotherapy

**Figure 5.**
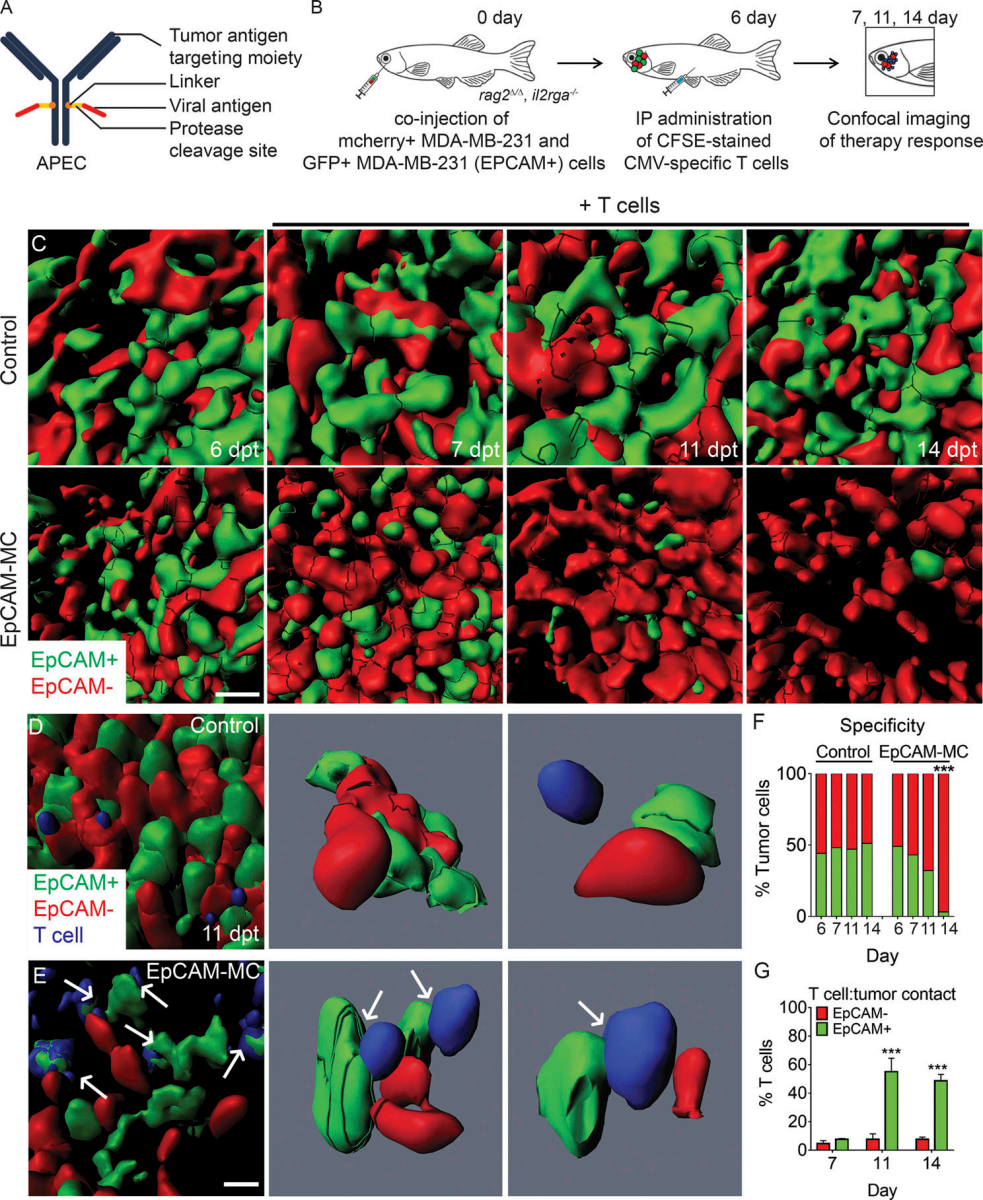
**Quantifying APEC immunotherapy responses and target specificity in human breast cancers in vivo.** **(A)** Schematic of APEC antibody. **(B)** Experimental design. EpCAM^−^/mCherry^+^ MDA–MB-231 breast cells compared with those engineered to express EpCAM (EpCAM^+^/GFP^+^). **(C)** 3D volumetric renderings of breast cancer cells engrafted into the periocular musculature (6 dpt, pretreatment) and following IP injection with CMV-specific T cells administered with either control EpCAM antibody (control) or EpCAM-MC (EpCAM). **(D and E)** High-magnification 3D modeling comparing location of T cells in control (D) and EpCAM-MC–treated animal (E) at 11 dpt. Arrows denote CFSE-stained T cells that directly contact GFP^+^ tumor cells. **(F)** Quantification of fluorescent tumor cell number before and after treatment (*n* > 566 tumor cells/time point, *n* = 5 animals/condition, 0.1 mm^3^ volume). **(G)** Percentage of T cells that directly contacted EpCAM^+^/GFP^+^ or EpCAM^−^/mCherry^+^ tumor cells (*n* = 5 animals/condition; error bars denote ±SD, 0.0125 mm^3^ volume). ***, P < 0.001, χ^2^ test (F) and Student’s *t* test (G). Scale bar equals 10 µm (C–E).

A high degree of target specificity is critical for any preclinical cancer immunotherapy currently in development. As such, we next investigated the specificity of the recently described APEC immunotherapy using *rag2^Δ/Δ^, il2rga^−/−^* zebrafish. APECs are antibody conjugates that deliver viral antigens to the tumor surface for presentation by HLA-I, specifically redirecting preexisting CD8^+^ T cell antiviral immunity against tumor cells following viral peptide cleavage by tumor proteases (Fig. 5 A; Millar et al., 2020). We first evaluated the efficiency of APEC immunotherapy by engrafting GFP-expressing EpCAM^+^ OVCAR-5 or EGFR^+^ MDA–MB-231 cells into the peritoneal cavity of *rag2^Δ/Δ^, il2rga^−/−^* mutant animals. Engrafted animals were then injected with CMV-specific CD8^+^ T cells along with (i) EpCAM control antibody, (ii) EpCAM-MMP7-CMV APEC (EpCAM-MC; unpublished data), or (iii) EGFR-MMP14-CMV APECs (EGFR-M14C) on days 7 and 14 after engraftment. EpCAM-MC and EGFR-M14C were generated using therapeutic EpCAM antibody (clone B38.1) and cetuximab, respectively. In particular, EpCAM-MC is a personalized APEC-immunotherapy for ovarian cancer that cleaves a CMV peptide (NLVPMVATV; abbreviated NLV) by tumor-expressed MMP7 and then is presented by MHCI (HLA-A*02:01). EpCAM-MC was identified as the top APEC that kills ovarian carcinoma in a xenograft screen that used high content imaging of therapy responses in *rag2^Δ/Δ^, il2rga^−/−^* zebrafish (unpublished data). Based on these successes, we also engineered a novel EGFR-M14C that contained the same NLV-CMV peptide but used the MMP14 cleavage. MDA–MB-231 cells express high levels of MMP14, which has important roles in driving tumor growth, invasion, and angiogenesis in mouse xenograft studies (Devy et al., 2009). Robust tumor regressions were only observed in animals that received CMV NLV peptide-primed, MHCI-restricted CD8^+^ T cells along with their target-specific APECs, while control antibody or nontargeted APECs failed to curb overall tumor growth in either model (Fig. S4, I–R). Like CAR T cells and BiTEs, our results show that APECs have exquisite specificity for killing only epitope-expressing tumors.

Building on these results, we next developed a colorimetric specificity assay to directly quantify on-target cell killing at single-cell resolution and to determine the possibility for collateral killing of adjacent nonepitope-expressing cancer cells by APECs. It had been suggested that the cleaved CMV peptide might be taken up by adjacent, nonepitope-expressing cancer cells and presented on the cell surface to engage T cells, further enhancing tumor cell killing of heterogenous cell populations that lack epitope (Millar et al., 2020). Specifically, MDA–MB-231 cells were generated to express mCherry or to coexpress both EpCAM and GFP (Fig. 5 B). Cells were mixed at 50:50 ratios and engrafted into recipient animals. As expected, EpCAM^−^/mCherry^+^ and EpCAM^+^/GFP^+^ MDA–MB-231 cells grew at similar rates in engrafted animals when injected with both NLV peptide-primed, MHCI-restricted CD8^+^ T cells and control EpCAM antibody (Fig. 5, C and F). By contrast, recipient animals that received CD8^+^ CMV-specific T cells and EpCAM-MC APEC exhibited significant decreases in EpCAM^+^/GFP^+^ cells over time, while EpCAM^−^/mCherry^+^ tumor cell number was largely unaffected (Fig. 5, C and F). Higher-magnification 3D modeling confirmed CMV-specific T cell interactions were far more frequent with EpCAM^+^/GFP^+^ tumor cells, but less so with EpCAM^−^/mCherry^+^ cells at 11 dpt (P < 0.001, Student’s *t* test; Fig. 5, D, E, and G; and Video 3). Similar results were also seen at 14 d after engraftment. Our work indicates that APEC therapy is highly specific to killing epitope-expressing cells and unexpectedly exhibits little effect on adjacent tumor cells that do not express the antigen.

**Video 3.** **3D modeling of APEC responses to MDA–MB-231 breast cancer cells grown in *rag2^Δ/Δ^, il2rga****^−/−^* **zebrafish.** Cells were engineered to express mCherry or to coexpress the EpCAM epitope and GFP. Control experiments used infusion of EpCAM antibody along with CMV-specific T cells (blue) and were compared with animals injected with EpCAM-MC APECs along with CMV-specific T cells (blue). Animals were imaged at 14 d after engraftment (8 d after infusion with T cell products). The control experiments show equivalent growth of GFP^+^ and mCherry^+^ MDA–MB-231 cells and minimal T cell interaction with tumor cells. By contrast, animals injected with APECs exhibited highly specific cell killing of EpCAM^+^/GFP^+^ tumor cells and have direct contact with CMV-specific T cells. 1× playback speed.

### Preclinical modeling of EGFR immunotherapies in RMS

**Figure 6.**
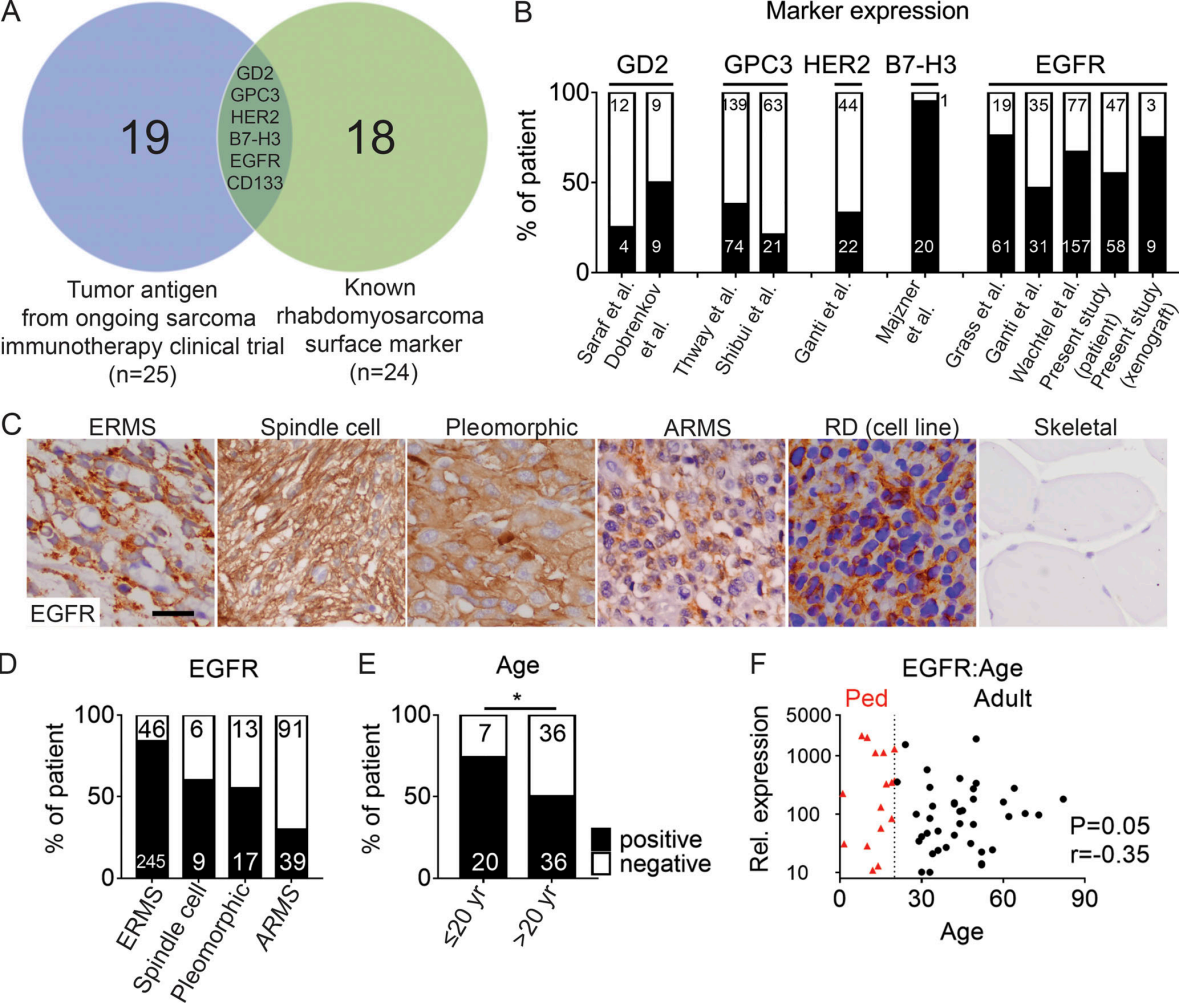
**EGFR is expressed in a large fraction of human RMS patients.** **(A)** Venn diagram showing overlap of RMS surface antigens that are currently being investigated in clinical trials for sarcomas. **(B)** Percentage of RMS patients expressing cell surface epitopes by IHC analysis. **(C)** IHC immunostaining of EGFR antibody on primary human RMS (four left panels), RD xenografts grown in NSG mice, and normal pediatric skeletal muscle. **(D)** Percentage of RMS patient samples expressing EGFR based on IHC staining across different subtypes of the disease. **(E)** Percentage of pediatric and adult RMS patients that express EGFR. *, P < 0.05, χ^2^ test. **(F)** Higher EGFR expression correlates with young age (P = 0.05, r = −0.35, linear regression analysis). Pediatric patients of <25 yr of age are shown by red triangles. Scale bar equals 10 µm (C). Ped, pediatric; Rel., relative.

Pediatric RMS is a common muscle cancer found in children and has an abysmal 5-yr survival rate of <30% in the metastatic, recurrent, and refractory disease settings (Yohe et al., 2019). Recently, two high-risk pediatric RMS patients were independently treated with HER2^+^ or CD56^+^ CAR T cells and achieved long-term disease remission, suggesting potential opportunities to exploit T cell immunotherapy in RMS (Hegde et al., 2020; Jiang et al., 2019). Despite these amazing success stories, the HER2 and CD56 receptors are only expressed in a small fraction of RMS patients (Bahrami et al., 2008; Ganti et al., 2006; Taniguchi et al., 2008). To systemically interrogate the most suitable antigen for this disease, we first identified all antigen targets that are currently being investigated in active sarcoma immunotherapy clinical trials and assessed if these markers are expressed in RMS (Fig. 6 A). In total, six surface antigens were identified as potential epitopes for RMS immunotherapy, including EGFR. A retrospective study using immunostaining of RMS patient samples (Dobrenkov et al., 2016; Ganti et al., 2006; Grass et al., 2009; Majzner et al., 2019; Saraf et al., 2019; Shibui et al., 2019; Thway et al., 2011) revealed EGFR expression in a substantial fraction of human RMS (*n* = 316 out of 476 primary patient samples and *n* = 9 of 12 cell line/PDXs; Fig. 6, B–D; and Table 1). Importantly, pediatric RMS patients had significantly higher incidence and overall expression levels of EGFR than patients with adult disease (74% vs. 50%, respectively, P = 0.03 by χ^2^ test; Fig. 6, E and F; and Table 1).

**Table 1.** Summary of RMS patient samples

	Disease subtype	Age	Male	Female	Total	Reference
Patient samples	Spindle cell RMS	Pediatric (<20 yr old)	3	0	3	Present study (Biomax SO2082a)
		Adult (>20 yr old)	11	1	12	Present study (Biomax SO2082a)
	Pleomorphic RMS	Pediatric (<20 yr old)	3	0	3	Present study (Biomax SO2082a)
		Adult (>20 yr old)	18	9	27	Present study (Biomax SO2082a)
	Embryonal RMS	Pediatric (mean age, 6.6 yr)	Unknown	Unknown	59	Grass et al., 2009
		Pediatric (mean age, 5.7 yr)	Unknown	Unknown	33	Ganti et al., 2006
		Pediatric (5–19 yr old)	Unknown	Unknown	173	Wachtel et al., 2006
		Pediatric (<20 yr old)	5	5	10	Present study (Biomax SO2082a)
		Adult (>20 yr old)	8	9	17	Present study (Biomax SO2082a)
	Alveolar RMS	Pediatric (mean age, 6.6 yr)	Unknown	Unknown	21	Grass et al., 2009
		Pediatric (mean age, 5.7 yr)	Unknown	Unknown	35	Ganti et al., 2006
		Pediatric (5–19 yr old)	Unknown	Unknown	47	Wachtel et al.
		Pediatric (<20 yr old)	5	5	10	Present study (Biomax SO2082a)
		Adult (>20 yr old)	8	9	17	Present study (Biomax SO2082a)
	Skeletal muscle (control)	Pediatric	0	1	1	Present study (Biomax SO2082a)
		Adult	7	1	8	Present study (Biomax SO2082a)
Cell line	Embryonal RMS	Pediatric	–	–	2	RD, SMS-CTR
	Alveolar RMS	Pediatric	–	–	2	Rh30, Rh41
PDX	Embryonal RMS	Pediatric	–	–	8	
	Alveolar RMS	Pediatric	–	–	4	

**Figure 7.**
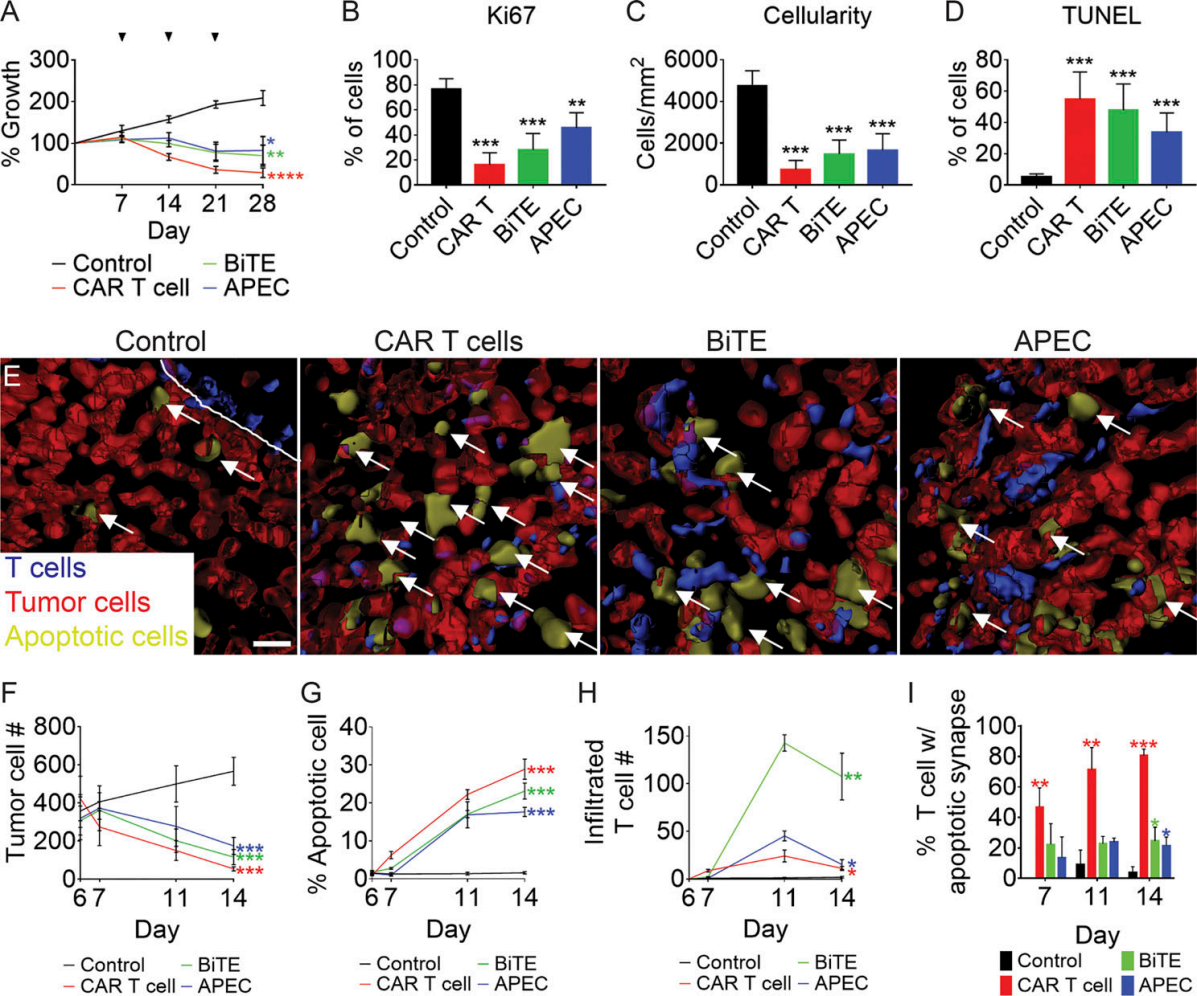
**EGFR-specific T cell immunotherapies for the treatment of RMS.** **(A–D)** *rag2^Δ/Δ^, il2rga^−/−^* animals engrafted by IP injection with EGFP^+^ RD cells and then coadministered weekly (i) control EGFR antibody along with CD8^+^ T cells, (ii) EGFR CAR T cells, (iii) EGFR/CD3 BiTE with CD8^+^ T cells, or (iv) EGFR-M14C with CMV-specific CD8^+^ T cells. Relative growth assessed by whole animal imaging with dosing noted by arrowheads (A). Quantification of proliferation by Ki67 IHC (B), cellularity based on H&E staining (C), and cell apoptosis by TUNEL (D) at 28 dpt. *n* = 3 animals/condition (B–D). **(E–I)** 3D modeling of T cell immunotherapy responses in animals engrafted with mCherry^+^/ZipGFP-Casp3^+^ RD cells into the periocular muscle imaged at 11 dpt. Control treated T cells (left, E) and T cell immunotherapy (right panels, E). Arrows denote representative examples of apoptotic tumor cells. Quantitation of therapy effects on tumor cell number (F; growth), tumor cell apoptosis (G), T cells infiltrated into tumor mass (H), and percentage of T cells in contact with apoptotic tumor cells (I). *n* = 5 animals/condition (A and F–I). *, P < 0.05; **, P < 0.01; ***, P < 0.001; ****, P < 0.0001, Student’s *t* test. Scale bar equals 10 µm (E). Error bars denote ±SD (A–D and F–I).

To investigate if and which T cell immunotherapy most effectively curbs RMS tumor growth in vivo, we next performed a side-by-side comparison of EGFR CAR T cells, BiTEs, and APECs to assess differences in killing of RMS cells engrafted into *rag2^Δ/Δ^, il2rga^−/−^* zebrafish. These experiments are the first to directly compare these three immunotherapy responses in vivo using the same tumor models and epitopes, allowing direct comparison of cell behavior and killing across immunoncology platforms. Specifically, 5 × 10^5^ RD cells were IP injected into *rag2^Δ/Δ^, il2rga^−/−^* mutant animals, and animals were administered (i) EGFR CAR T cells, (ii) EGFR/CD3 BiTE with CD8^+^ T cells, (iii) EGFR-M14C with CMV-specific T cells, or (iv) control EGFR antibody with CD8^+^ T cells on days 7, 14, and 21 after engraftment (*n* = 5 animals/arm; Fig. 7 A). Notably, significant reductions in tumor burden were observed in all three immunotherapies tested, with EGFR CAR T cells eliciting a trend toward more potent cell killing by 21 d after therapy that was associated with reduced Ki67 proliferation marker expression, higher numbers of TUNEL^+^ apoptotic cells, and subsequently fewer tumor cells per area on section (Fig. 7, A–D).

To further understand the kinetics of cell killing and possible differences in T cell infiltration and engagement between the three therapies, we next engrafted 5 × 10^4^ ZipGFP-Casp3–expressing RD cells into the periocular muscle and used confocal imaging to quantify T cell infiltration and cell apoptosis following administration of each T cell immunotherapy (Fig. 7, E–I). Among the three therapies analyzed, EGFR/CD3 BiTEs induced the most robust T cell infiltration (Fig. 7, E and H). Yet despite fewer CAR T cells entering the tumor, they elicited the most effective tumor cell killing and subsequent reductions in tumor cell number over time (P < 0.02, Student’s *t* test; Fig. 7, F and G), suggesting higher overall therapy efficacy of CAR T cell immunotherapies. Indeed, high-resolution 3D modeling of T cell–tumor cell interaction revealed a higher percentage of T cells could induce tumor cell killing by CAR T cells compared with BiTE- and APEC-treated animals (*n* = 5 animals/arm; P < 0.01, Student’s *t* test; Fig. 7 I). Finally, although APEC-treated cells had less infiltrative capacity to enter tumors (Fig. 7 H), both BiTEs and APECs had similar potency in killing tumor cells following infiltration into the tumor mass (Fig. 7 I).

**Figure 8.**
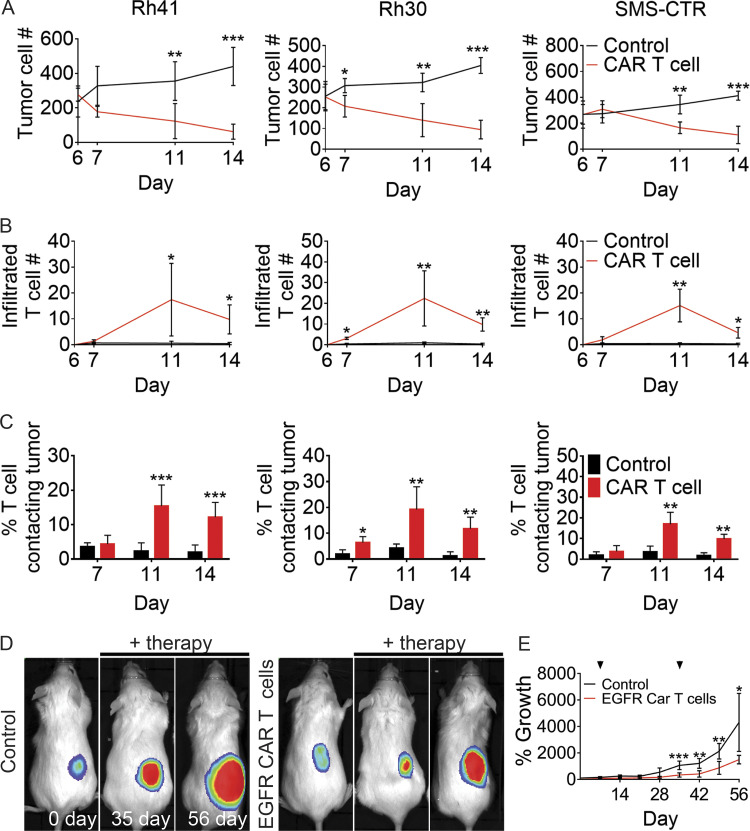
**EGFR CAR T cells kill human RMS cells in both *rag2^Δ/Δ^, il2rga****^−/−^* **zebrafish and NSG mouse xenograft models.** **(A)** Quantification of total tumor cell number within each experimental animal (*n* = 5 fish/experimental arm). **(B)** Quantification of CAR T cells infiltrated into the tumor mass over time (*n* = 5 animals/experimental arm). **(C)** Percentage of T cells that directly contacted RMS tumor cells. **(D)** Representative luciferase images of RD xenografted tumors in NSG mice (*n* = 6 mice/experimental arm). **(E)** Quantification of relative growth of xenografted RD tumors in NSG mice receiving either control CD8^+^ T cells or EGFR CAR T cells. Arrowheads denote time of CAR T cell administration. *, P < 0.05; **, P < 0.01; ***, P < 0.001, Student’s *t* test. Error bars denote ±SD (A–C and E). Rh41 and Rh30, fusion-positive RMS; SMS-CTR, fusion-negative RMS.

We next confirmed EGFR CAR T cell responses in a wider array of RMS tumors including both fusion-positive and fusion-negative subtypes of RMSs. As was seen in RD RMS xenografted cells, EGFR CAR T cells efficiently infiltrated into the tumor and effectively killed RMS tumor cells over time in three additional xenograft models irrespective of disease subtype (*n* = 5 zebrafish/arm; P < 0.006, Student’s *t* test; Fig. 8, A–C). As was seen in other CAR T cell indications tested in our work, there was an overall lower percentage of CAR T cells engaged with tumor at any given time compared with BiTE and APEC treatment. Yet these CAR T cell therapies efficiently killed tumors over longer periods of time. These results again highlight differences in the rates of cell killing by these modalities. Importantly, EGFR CAR T cell immunotherapy was also effective in suppressing RMS xenograft growth in NSG mice (*n* = 6 mice/arm; P < 0.05 over multiple time point analysis; Fig. 8 D). Together, our zebrafish and mouse xenograft studies provide much-needed preclinical rationale for assessing a wide array of EGFR-targeted immunotherapies in RMS.

## Discussion

Our work has established the *rag2^Δ/Δ^, il2rga^−/−^* zebrafish as a powerful model for assessing T cell immunotherapy responses at single-cell resolution, successfully imaging single-cell therapy responses in 10 cancer xenograft models across eight distinct T cell immunotherapies. Recently, others have leveraged the zebrafish larval cell transplantation platform to model the effects of tumor-infiltrating lymphocytes and CD19 CAR T cells in human cancer xenografts (He et al., 2020; Pascoal et al., 2020). Yet, these larval xenotransplant studies were limited to engraftment of only a few hundred cells per animal, animals could not be reared at physiological 37°C, and assay times were restricted to 24 h in both studies. By contrast, our studies demonstrated the utility of the adult immunocompromised zebrafish model in a wide array of immunotherapies including CAR T cells, BiTEs, and APECs. We also optimized intravital imaging approaches to quantitatively assess T cell migration and infiltration into the tumor mass and tumor cell killing in real time and document target specificity serially over days. Our studies also demonstrated that most efficient T cell immunotherapy–induced cell killing falls between 24 and 96 h after administration, time points that would not be evaluable using the larval xenograft models.

The adult immune-deficient zebrafish model is more akin to xenograft studies that use NSG mice, where longer time course studies allow for assessment of immune-therapy responses for up to 3 wk. Mouse xenograft studies have led to many important insights into mechanisms of T cell homing, infiltration, and tumor killing (Boissonnas et al., 2007; Boulch et al., 2021; Cazaux et al., 2019; Halle et al., 2016; Hoekstra et al., 2020; Mulazzani et al., 2019). Yet, the zebrafish xenograft model addresses several fundamental difficulties of completing similar studies using mouse xenografts. For example, imaging T cell/tumor cell responses in mouse xenografts is more technically challenging and requires complex multi-photon microscopy and prior knowledge of where to image based on surgical window creation. Mice are also more expensive; thus, large-scale studies are limited. Despite the perceived powers of the zebrafish xenograft model, it also has limitations. For example, xenografted zebrafish were unable to be imaged longer than 10 min in our studies, mainly attributed to gill respiration movements around the periocular engraftment site, precluding a more detailed time-lapse study of T cell movement and subsequent tumor cell killing. These technical hurdles will likely be addressed by the development of deep gill-perfusion anesthesia techniques, selection of alternative engraftment sites such as the dorsal musculature, and refined imaging platforms in the future.

Despite the many advantages of engrafting human tumors and assessing immunotherapy responses in adult *rag2^Δ/Δ^, il2rga^−/−^* zebrafish and NSG mice, both models lack endogenous T, B, NK, and macrophage cells, precluding assessment of how these important immune cells modulate the tissue microenvironment and modify responses to T cell therapy. New immune-deficient zebrafish models will likely incorporate transgenic approaches to express human cytokines that support human blood cells and allow for further humanization, akin to knock-in and transgenic mouse models such as the humanized M-CSF^h/h^, IL-3/GM-CSF^h/h^, SIRPa^h/h^, TPO^h/h^, RAG2^−/−^, IL2Rg^−/− ^mice (Das et al., 2016; Rongvaux et al., 2014; Wunderlich et al., 2010; Wunderlich and Mulloy, 2016). Such models would permit engraftment of both the human immune system and tumor to follow more complex immune cell interactions. In fact, elegant work from the Berman group has already developed transgenic fish that express human *GM-CSF*, stem cell factor, and stromal derived factor 1α and permit short-term engraftment of human hematopoietic stem cells and leukemia cells into larval fish, providing an important starting point for such studies (Rajan et al., 2020).

At a cell biological level, our work demonstrated important differences in early responses between CAR T cell, BiTE, and APEC immunotherapies. For example, single-cell quantitative studies revealed that CAR T cells engaged with tumor cells by just 24 h after engraftment in most xenograft models, while neither BiTE- nor APEC-treated T cells exhibited such robust early responses. CAR T cell–tumor cell interactions also led to elevated overall tumor cell killing as read out by an overall reduction in tumor cell number and higher percentage of apoptotic synapse formation between CAR T cell–tumor cells compared with BiTE- and APEC-treated T cells. Although our work showed a direct correlation between T cell/tumor cell contact and killing, recent work has also demonstrated bystander/indirect killing trough cytokines secreted by T cells in the absence of direct contact between tumor cell and T cells, suggesting multiple possible modes of tumor cell killing (Hoekstra et al., 2020). In total, differences in T cell engagement and cytotoxicity observed in our work are likely accounted for by the fundamentally different mechanisms by which CAR T cells, BiTEs, and APECs bind to tumor cells, induce activation, and kill. For example, CAR T cells engage tumor cells through a chimeric antigen receptor scFv domain and are primed for activation by ex vivo stimulation (Rafiq et al., 2020); thus, robust and fast tumor cell killing would be expected. T cell receptor/antigen binding then activates CAR T cells, releasing perforin, granzyme, and apoptosis-inducing cytokines. In contrast, BiTEs mechanically link nonstimulated T cells with tumor cells by simultaneously binding a tumor-specific antigen and the CD3 receptor on T cells (Huehls et al., 2015). Antibody/CD3 binding then activates T cells, eliciting endogenous T cell effector responses. Finally, APECs harness the antiviral CD8^+^ T cell responses by loading viral epitopes onto tumor-expressed MHCI, promoting the formation of MHC/TCR complexes with viral-specific T cells and subsequent activation (Millar et al., 2020). The processes by which BiTEs and APECs induce T cell activation would be predicted to require longer interaction times between the T cell with tumor before inducing cell death compared with CAR T cells. In total, our observations are largely mirrored by in vivo mice xenograft studies targeting NY–ESO-1 antigen on human myeloma cells or 237scFV on mice fibrosarcoma cells (Maruta et al., 2019; Stone et al., 2012). These studies performed a side-by-side comparison of CAR T cell and BiTE cell killing efficacy using mouse models and in both instances showed CAR T cells to be superior at killing tumor cells over long–time course experiments. However, these murine models lacked the resolution to visualize tumor cell–immune cell interactions at single-cell resolution and hence failed to provide mechanistic insights into the early kinetic differences between T cell–based therapies and linking of these cell biological differences with effects on tumor cell killing.

Our work also validated the efficacy and target specificity of the new APEC technology to kill antigen-expressing tumor cells. First, we demonstrated the feasibility and in vivo utility of designing APECs that express tumor-specific protease cleavage sites in the context of ovarian carcinoma (unpublished data). This EpCAM-MC APEC was created by conjugating an MMP7 protease cleavage site (AVSRLRAYNLVPMVATV) with a CMV-specific viral epitope NLV to the therapeutic EpCAM antibody (Clone 38.1; unpublished data). Because APECs bind to tumor-specific epitopes and only release their viral peptide cargo locally following cleavage by tumor-secreted proteases, target specificity is expected to be enhanced over other T cell immunotherapies, including traditional CAR T cells and BiTEs. APEC approaches thus allow exquisite redirection of endogenous viral CD8^+^ T cells to kill seemingly virally infected tumor cells. Here, we validated the remarkable specificity for epitope-expressing tumor cells with little collateral killing of nonepitope-expressing tumor cells. Building on these successes, we also developed a new EGFR-M14C APEC that efficiently killed RMS cells. In total, our work has shown that APECs are a new and powerful immunotherapy that will be valuable for tailoring personalized immunotherapy, pairing tumor-specific epitope expression with tumor-specific protease expression and cleavage.

Our work also has established much needed preclinical modeling for T cell–mediated immunotherapy in RMS and identified EGFR-based immunotherapies as a new approach to kill RMS tumor cells. EGFR is expressed in a large fraction of RMS patients, and clinical trials using EGFR immunotherapies have reported tolerable side effects (Guo et al., 2018). Despite achieving remissions in a large fraction of pediatric RMSs, relapse, refractory, and recurrent metastatic diseases are the major clinical challenge facing patients. Current treatment strategies rely on aggressive treatment that includes radiation, chemotherapy, and surgery (Yohe et al., 2019). Targeted immunotherapy approaches could prove powerful for the treatment of aggressive RMS tumors, especially in the relapse setting and for those tumors that cannot be easily resected. Indeed, two pediatric RMS patients with refractory metastatic and recurrent disease achieved complete remissions following HER2^+^ and CD56^+^ CAR T therapy (Hegde et al., 2020; Jiang et al., 2019), suggesting a clinical path forward for T cell therapies in this disease. Our zebrafish and mouse xenograft studies have provided a strong preclinical rationale for assessing EGFR CAR T cell and other immunotherapies in this disease.

## Materials and methods

### Animal welfare assurances and husbandry

All animal studies were approved by the Massachusetts General Hospital subcommittee on research animal care under protocols #2011N000127 (zebrafish) and #2013N000038 (mouse) and by the Partners human research committee under institutional review board protocol #2009P002756. All immunocompromised zebrafish and mice used in this study were kept in BCL2 animal facilities, with regular veterinary checks. Husbandry and rearing of adult immune-compromised zebrafish were completed essentially as previously described (Yan et al., 2019; Yan et al., 2020). Our protocol deviated only slightly based on feeding engrafted fish three times daily with 100 mg/animal of GEMMA micro300 supplemented with cell line–specific culture media (ratio 1:4), 10% FBS, and 1% penicillin-streptomycin-glutamine.

### Generating *rag2^Δ/Δ^* gene deletion and compound mutant zebrafish

*rag2^Δ/Δ^* zebrafish were created in the Casper-strain zebrafish using CRISPR/Cas9 genome editing (Hwang et al., 2013). Briefly, two guide RNA (gRNA) sequences (5′-AGA​ACC​GTA​TCA​AGC​GCG​GG-3′ and 5′-GGC​CCT​TGA​CTA​CAT​ATG​GTG-3′) were designed to flank the 3.3 kb of the rag2 gene locus using CHOPCHOP. gRNAs were cloned into the DR274 vector (Addgene) and transcribed in vitro using the T3 mMESSAGE mMACHINE Kit. A mixture of 300 ng/µl of Cas9 mRNA and 100 ng/µl of gRNA was coinjected into one cell–stage zebrafish embryos. F0 animals were incrossed, and progeny were assessed for gene deletion using PCR of genomic DNA. WT gene-specific primers amplified a 444-bp fragment of the endogenous rag2 gene (forward 5′-CCA​TAT​AGC​CAA​TTA​CTA​C-3′, reverse 5′-GCA​GAT​CTG​GAT​CTG​GAG​T-3′; denaturing: 94°C/30 s, annealing: 60°C/30 s, elongation: 68°C/60 s, and termination: 68°C/5 min). Mutant gene–specific primers (forward 5′-CCC​ATC​TAT​GGG​AAA​CTA​TC-3′, reverse 5′-GTG​TCA​CAT​GAT​CCT​TCA​G-3′) spanned the genomic deletion and amplified a 973-bp fragment (denaturing: 94°C/30 s, annealing: 60°C/30 s, elongation: 68°C/60 s, 36 cycles, termination: 68°C/5 min; Fig. S1, A–C). Il2rgaY91fs-specific primers (forward 5′-TTT​GAC​ATC​GAA​GAC​TGT​CCT​G-3′, reverse 5′-GTC​CTG​TAA​CGA​ACT​TCG​CTC​T-3′) spanned the genomic mutation, amplifying a 373-bp WT allele and a 360-bp mutant allele (denaturing: 94°C/30 s, annealing: 60°C/30 s, elongation: 68°C/30 s, 36 cycles, and termination: 68°C/5 min). Male *rag2^Δ/Δ^, Il2rga^Y91fs/+^* adult Casper (double mutant for roy^a9/a9^ and nacre^w2/w2^) zebrafish were crossed to female *rag2^Δ/+^, il2rga^Y91fs/+^* adult Casper zebrafish. Progeny were grown to adulthood and then subjected to scale resection genotyping at 2–3 mo of age as previously described (Yan et al., 2019). Homozygous mutant *rag2^Δ/Δ^, il2rga^−/−^* fish were produced at that expected Mendelian ratio (11.75%, *n* = 984 of 8,374 fish, expected 12.5%).

### Single-cell RNA sequencing and analysis of immune cell defects in *rag2^Δ/Δ^, il2rga^−/−^* fish

InDrop single-cell sequencing was completed on adult zebrafish kidney marrow as previously described (Tang et al., 2017). Briefly, 2-mo-old zebrafish were sacrificed, marrow extracted, and made into single-cell suspension for microfluidic encapsulation. Library construction was performed by the Harvard Institute of Chemistry and Cell Biology Single Cell Core using methods outlined in Klein et al. (2015). Libraries were sequenced using the Nextseq 500 High Output V2 Kit. T-distributed stochastic neighbor embedding (tSNE) visualization used combined samples from three fish per genotype (WT, *n* = 4,654 cells; *rag2^Δ/Δ^*, *n* = 9,418 cells; and *rag2^Δ/Δ^, il2rga^−/−^*, *n* = 8,790 cells).

### Human cell lines, PDXs, and authentication

All human cell lines used in this work were authenticated just before use by small tandem repeat profiling using the Whatman Flinders Technology Associates sample collection kit (American Type Culture Collection [ATCC]). Briefly, cells were diluted to 10^6^ cells/ml. 40 µl of cell suspension was used to spot on to FTA blotting paper. FTA sample collection kits were then submitted to ATCC for authentication by small tandem repeat profiling. Cell lines were cultured according to ATCC’s recommendations, supplemented with 10% FBS (Atlanta Biologicals) and 1% penicillin/streptomycin/glutamine (Life Tech), and grown at 5% CO_2_ at 37°C. Adherent cells were dissociated using 0.025% trypsin for 2 min. Adherent cell PDX models used in this work included MGH1518 and MGH1528 (Drapkin et al., 2018; Farago et al., 2019). Both PDXs were stained with 1 µM ViaFluor SE cell proliferation stain (Biotium) before transplantation according to manufacturer’s protocol.

### Lentiviral vectors and creation of stable cell lines

2 µg of pLenti-CMV-GFP-puro was transfected into HEK293T cells with 2 µg pCMV-dR8.91, 0.2 µg pVSV-g, and TransIT-LT1 reagent (Mirus Bio). Supernatants containing the lentivirus were collected, filtered, and added to the cell lines used in this study in the presence of 4 µg/ml polybrene (Millipore). Viral particle containing pLenti-CMV-GFP-puro and pLV-mCherry was added to cell lines in a subset of our studies. 1 µg/well of pZipGFP-Casp3 was added to RD or OVCAR-5 cells (3 × 10^5^ cells/well in a 6-well plate). RD or OVCAR-5 transfected cells were pooled, FACS selected for fluorescent cells, and replated to obtain stable-expressing cell lines.

### T cell expansion, CAR T cell, and NLV-specific T cell production

CD8^+^ T cells used as control for BiTE experiments were purchased (Stem Cell Technology; #70027), cultured, and expanded using RPMI medium (10% FBS and 1% penicillin/streptomycin) supplemented with 20 IU/ml of rhIL-2.

CAR T cells were generated using primary donor T cells transduced with the anti-CD19 or EGFRvIII CAR containing a 4-1BB intracellular signaling domain and expanded as previously reported (Milone et al., 2009). Briefly, donor T cells were thawed and activated using α-CD3/α-CD28 Dynabeads (Life Technologies) at a 1:3 T cell:beads ratio. The cells were cultured in RPMI medium (10% FBS and 1% penicillin/streptomycin) supplemented with 20 IU/ml of rhIL-2. Every 2 d, fresh medium was added to keep the cells at a concentration of 0.5–2 × 10^6^/ml. For CAR T cell production, lentiviral vector was added to the culture 24 h after activation. In parallel, donor-matched T cells that had been activated but untransduced were expanded to serve as a negative control in subsequent experiments. At days 12–14 of culture, CAR expression was determined.

NLV-specific T cell lines were created from CMV-seropositive peripheral blood mononuclear cells isolated from healthy donors upon stimulation with 10 μg/ml CMV peptide HLA-A*02:01 restricted NLV (Genscript) in RPMI1640 supplemented with 10% FBS, 100 U/ml each of penicillin and streptomycin, 4 mM of L-glutamine, and 1% human AB serum (Sigma). On day 4, half culture medium was replaced with the same medium plus 500 IU/ml IL-2 (Peprotech) and changed twice weekly. NLV-specific CD8^+^ T cells were identified by flow cytometry with staining of HLA–peptide tetrameric complex on day 14–21. Only those T cell lines with >50% NLV-specific CD8^+^ T cells were qualified for the next functional assay for a maximum of 6 wk.

### APEC production

Antibody was conjugated with peptide as previously described (Millar et al., 2020). Briefly, therapeutic anti-EpCAM (clone B38.1) or cetuximab (anti-EGFR) was reduced by 10 mM Tris(2-carboxyethyl) phosphine, and MMP7 (EpCAM; AVSRLRAYNLVPMVATV) or broad reactive MMP14 (EGFR; PRSAKELRNLVPMVATV) cleavable peptide containing an N-terminal 3-maleimido propionic acid group was added for antibody and peptide conjugation. Unbound, free peptide was quenched with 10 mM N-acetyl cysteine and removed using the Pur-A-Lyzer Midi Dialysis Kit (Sigma; PURD 35010). Conjugated anti-EpCAM (clone B38.1) and anti-EGFR were diluted to the required concentration in PBS.

### Human cancer cell transplantation into zebrafish and quantifying tumor growth

Human cancer cell lines were transplanted into *rag2^Δ/Δ^, il2rga^−/−^* as previously described (Yan et al., 2019). Human tumor cells were transplanted into either the IP cavity or periocular musculature. Briefly, cells were grown to 90% confluence in T75 cell culture flasks, trypsinized if adherent, counted, and only used for transplantation when viability was >90%. 5 × 10^5^ cells/fish were injected into the peritoneal cavity of recipient fish using a 30 1/2–G needle (BD; 10 µl of volume used for transplantation). For ocular muscle injections, 3–5 µl of cell suspension was injected (5 × 10^4^ cells/fish). Recipient zebrafish were then raised at 37°C in antibiotic-supplemented fish water. Epifluorescent whole-animal imaging of tumor burden was performed under constant UV light intensity and camera exposure (microscope: Olympus MVX10; camera: Olympus DP74). Tumor volume was determined by quantification of average fluorescence intensity multiplied by tumor area using ImageJ. Single-cell resolution imaging of periocular engrafted cells was performed using confocal microscopic imaging at 100× (Zeiss LSM710 inverted microscope; see below). Maximum projection images were created from 100-micron stacks (10 microns per confocal slice) and quantified using Imaris. Recipient fishes were sacrificed at the end of each experiment, fixed in 4% paraformaldehyde, and sectioned for histological examination as previously described (Yan et al., 2019). Similar approaches were used for engraftment of cancer cells labeled with other fluorescent-reporters and dyes.

### Live zebrafish confocal single-cell imaging and analysis

Imaging of periocular transplanted cells was performed using an inverted LSM 710 confocal microscope (Zeiss) with Zen software platform (Zeiss), as previously described (Yan et al., 2019; Yan et al., 2020). Engrafted zebrafish were anesthetized using low-dose 0.01% tricaine (Western Chemical), placed onto a 36-mm glass-bottom dish (Thermo Fisher Scientific; #150680), and the fish torso was embedded in 1% low–melting point agarose (Thermo Fisher Scientific) to stabilize the animal for imaging. To keep the animal under anesthetic during imaging, zebrafish were submerged in 5 ml of warm 37°C fish water containing 0.01% tricaine. Serial z-stack imaging was performed using a 10× objective (numerical aperture, 0.45), achieving an overall 100× magnification. GFP- and mCherry-expressing engrafted animals were imaged using the 488-nm (emission = 493–586 nm) and 546-nm laser (emission = 575–703 nm). All T cells used in this study were stained with 1 µM of ViaFluor CFSE before transplantation and imaged using a 405-nm laser (emission = 350–470 nm). In vivo cell apoptosis of ZipGFP-Casp3 imaging studies were imaged used a 488-nm (emission = 493–586 nm) and 546-nm laser (emission = 575–703 nm).

Images were automatically annotated and counted in Imaris. Total T cell, tumor cell, and apoptotic cell (ZipGFP-Casp3^+^) numbers were quantified using Imaris spot and surface functions. Quantification was completed on z-stack images with dimensions 1,000 µm × 1,000 µm × 100 µm (0.1 mm^3^). 3D modeling analysis quantifying absolute distance between T cells and tumor cells with direct cell-to-cell contact was defined by <10 µm between cells. This analysis was completed on z-stack tumor images sampled at 500 µm × 500 µm × 50 µm (0.0125 mm^3^) and analyzed using the distance transformation, distance between spot to surface, and spot close to surface XTension functions. Apoptotic cells were pseudo-colored yellow using Imaris surface function for easy visualization in Fig. 7 E.

### Assessing immunotherapy responses in zebrafish xenografts

Fluorescent-labeled human cancer cell lines (U87, JeKo-1, OVCAR-5, K562, MDA–MB-231, RD, SMS-CTR, Rh41, and Rh30) were transplanted IP (5 × 10^5^ cells/animal) or periocularly (5 × 10^4^ cells/animal) into recipient fish. Stably engrafted fish were IP injected with CAR T cells (5 × 10^5^ cells/dose). For BiTE experiments, animals were coinjected with 5 × 10^5^ CD8^+^ T cells along with control antibodies (EpCAM, 50 µg/kg; CD19, 50 µg/kg, or EGFR, 50 µg/kg), solitomab (50 µg/kg), blinatumomab (250 µg/kg), or EGFR/CD3 BiTE (10 µg/kg). For APEC experiments, animals were injected with 5 × 10^5^ CMV-specific T cells, along with control antibodies (EpCAM, 50 µg/kg, or EGFR, 50 µg/kg), EpCAM-MC (50 µg/kg), or EGFR-M14C (50 µg/kg). All IP injections were completed using a 30 1/2–G needle (BD) at the time points noted for specific experiments. At the end of the experiment, animals were fixed in 4% paraformaldehyde, sectioned, and examined histologically by H&E staining, immunohistochemistry (IHC) for Ki67, and TUNEL. Comparisons between groups were performed using ANOVA followed by Student’s *t* test.

### Assessing olaparib and temozolomide responses in zebrafish xenografts

CFSE-stained MGH1518 and MGH1528 cells were transplanted IP, and engrafted fish were orally gavaged with 10 µl of drug at 50 mg/kg of olaparib and 25 mg/kg of temozolomide or vehicle control (1% DMSO in 1× PBS). Gavage was performed using a Hamilton 22-G needle and 22-G soft-tip catheter tubing (Yan et al., 2020). Drugs were orally administered at 7 dpt, and recipient fishes were imaged as outlined above. At 14 d, animals were fixed in 4% paraformaldehyde, sectioned, and examined histologically. Comparison between groups was performed using Student’s *t* test (*n* = 5 fish/treatment arm).

### Histology and IHC evaluation

Engrafted zebrafish were fixed in 4% paraformaldehyde, embedded in paraffin, and sectioned at 5-mm thickness. Sections were stained by H&E, IHC, or immunofluorescence. For IHC staining, the primary antibodies were rabbit monoclonal anti-Ki67 (1:1,000 dilution; Abcam), monoclonal anti-CD3 (1:100 dilution; Abcam), monoclonal anti-GFP (1:100 dilution; Abcam), monoclonal anti-EGFR (1:100 dilution; Abcam), and TUNEL (1:1,000 dilution; Thermo Fisher Scientific). Secondary antibodies were biotinylated goat anti-rabbit IgG antibody (Vectorlabs) and horse anti-mouse antibody (Vectorlabs). Development was completed using Vectastain ABC Kit (Vectorlabs) or Alexa Fluor 488 anti-mouse and Alexa Fluor 546 anti-rabbit secondary antibody (1:1,000 dilution for all secondary antibodies).

Quantification was completed based on counting three randomly selected fields imaged at 400× using an Olympus BX41 microscope. Quantification used ImageJ and was blinded. Images were counted without labels by Eric Alpert. Samples were analyzed using a fixed threshold for achieving an unbiased, quantitative assessment of the IHC and TUNEL staining within the selected imaged field (Yan et al., 2020). Percentage of proliferating (Ki67) and apoptotic (TUNEL) cells was calculated by dividing the number of positively stained cells by the total number of cells counted within the selected fields. *n* > 200 control cells were analyzed per sample, with fewer cells being counted for treated samples. H&E-stained sections were imaged at 400× and quantified as number of cells per unit area.

### Human cancer cell transplantation into mice

3 × 10^6^ luciferase-expressing RMS RD cells were embedded into Matrigel at a 1:1 ratio and injected subcutaneously into the flank of 8-wk-old female anaesthetized NSG mice (Charles River Laboratories). When engrafted tumor reached a volume of 300 mm^3^, two doses of 3 × 10^6^ CAR T cells were administered on days 0 and 35 of treatment, respectively, by tail vein injection. Tumor growth was quantified by overall luciferase bioluminescent imaging using the IVIS imaging system once a week.

### Quantification and statistical analysis

Statistical details, including *n* values, P values, and statistical tests, are detailed in the Materials and methods, Results, and figure legends. Data in bar graphs are shown as an absolute number with mean ± SD noted. ANOVA and Student’s *t* tests were used to calculate significant differences where indicated. A subset of experiments used the χ^2^ test to compare values across two samples. P ≤ 0.05 was considered statistically significant. In all experiments, zebrafish and mice were randomly assigned to experimental groups. All statistical analysis were performed using Prism 7 (GraphPad).

### Online supplemental material

Fig. S1 shows creation, genotyping, and viability of *rag2^Δ/Δ^, il2rga^−/−^* immunocompromised zebrafish. Fig. S2 shows engraftment of human cancer cells into *rag2^Δ/Δ^, il2rga^−/−^* zebrafish. Fig. S3 shows *rag2^Δ/Δ^, il2rga^−/−^* zebrafish to accurately predict olaparib and temozolomide therapy responses in PDXs of small cell lung cancer. Fig. S4 shows preclinical evaluation of CAR T cell, BiTE, and APEC immunotherapies following IP engraftment of human cancers into *rag2^Δ/Δ^, il2rga^−/−^* zebrafish. Fig. S5 shows IHC validation of T cell infiltration into engrafted tumors following immunotherapy. Table 1 lists RMS patients for data rendered in Fig. 6. Video 1 shows a 3D modeling of CAR T cell responses to U87-EGFRvIII glioma tumors grown in a *rag2^Δ/Δ^, il2rga^−/−^* zebrafish. Video 2 shows 3D modeling of BiTE responses to OVCAR-5 ovarian carcinoma cells grown in *rag2^Δ/Δ^, il2rga^−/−^* zebrafish. Video 3 shows 3D modeling of APEC responses to MDA–MB-231 breast cancer cells grown in *rag2^Δ/Δ^, il2rga^−/−^* zebrafish.

## Data Availability

Data deposition for InDrops sequencing results is available in the Gene Expression Omnibus database (accession no. GSE179401).
